# Gut Microbiota, Immunity, and Metabolism in the Progression From Chronic Liver Disease to Hepatocellular Carcinoma

**DOI:** 10.1002/advs.202523582

**Published:** 2026-07-10

**Authors:** Yi Hu, Caiyan Lin, Lijie Zhang, Xue Jiang, Huawen Li, Yangzhe Wu

**Affiliations:** ^1^ Immunology Department School of Medicine Jinan University Guangzhou Guangdong P. R. China; ^2^ Key Laboratory of Viral Pathogenesis & Infection Prevention and Control (Jinan University) Ministry of Education Guangzhou P. R. China; ^3^ College of Bioengineering Zunyi Medical University Zhuhai Guangdong P. R. China; ^4^ Gynecology Department Zhuhai People's Hospital (Zhuhai Clinical Medical College of Jinan University, The Affiliated Hospital of Beijing Institute of Technology) Zhuhai Guangdong P. R. China; ^5^ Clinical Center for Immunity and Gut Microbiota Zhuhai People's Hospital (Zhuhai Clinical Medical College of Jinan University, The Affiliated Hospital of Beijing Institute of Technology) Zhuhai Guangdong P. R. China; ^6^ Institute of Translational Medicine Zhuhai People's Hospital (Zhuhai Clinical Medical College of Jinan University, The Affiliated Hospital of Beijing Institute of Technology) Zhuhai Guangdong P. R. China; ^7^ Guangdong Provincial Key Laboratory of Tumor Interventional Diagnosis and Treatment Zhuhai People's Hospital (Zhuhai Clinical Medical College of Jinan University, The Affiliated Hospital of Beijing Institute of Technology) Zhuhai Guangdong P. R. China

**Keywords:** chronic liver disease, dietary interventions, gut microbiota, hepatocellular carcinoma, immunometabolism, microbial metabolites, precision nutrition

## Abstract

The progression from chronic liver injury to hepatocellular carcinoma (HCC) should be viewed as a heterogeneous continuum of immune, metabolic, fibrotic, and microbial remodeling rather than as a single linear route. Although this review uses the MASLD‐MASH‐fibrosis/cirrhosis‐HCC sequence as a mechanistically informative model, the gut‐liver‐immune framework is also relevant, with important etiology‐specific differences, to alcohol‐associated liver disease (ALD), chronic hepatitis B virus (HBV) infection, chronic hepatitis C virus (HCV) infection, and mixed‐etiology liver disease. Across these contexts, hepatocyte lipotoxicity or viral/alcohol‐induced injury, mitochondrial stress, endotoxemia, altered bile‐acid signaling, fibrotic remodeling, and immune exhaustion progressively reshape the hepatic microenvironment toward tumor‐permissive inflammation and immune escape. We integrate transcriptomic, single‐cell, spatial, microbial, and metabolomic evidence to define stage‐ and etiology‐dependent immunometabolic states. Particular emphasis is placed on microbial metabolites, including short‐chain fatty acids, secondary bile acids, and tryptophan‐derived indoles, which engage host receptors such as FFAR2/3, GPR109A, FXR, TGR5, AhR, and PXR to influence lipid metabolism, epithelial barrier integrity, cytokine programs, epigenetic remodeling, and antitumor surveillance. We further discuss how sex, baseline microbiome composition, hepatic zonation, and preclinical model selection influence disease trajectories and therapeutic responses. By focusing on the gut microbiota‐metabolism‐immunity axis, this review provides a systems‐level framework for biomarker discovery, risk stratification, precision nutrition, and rational combination therapies. Targeting the coordinated interplay among diet, microbiota, metabolism, immunity, and the hepatic spatial niche may help intercept chronic liver disease before malignant transformation and improve therapeutic responses in established HCC.

## Graphical abstract

This review explains how chronic liver injury progresses toward hepatocellular carcinoma through interconnected changes in gut microbes, metabolism, immunity, fibrosis, and diet. It highlights microbial metabolites, bile‐acid signaling, immune dysfunction, and nutritional or microbiome‐based interventions as opportunities to identify risk earlier, interrupt disease progression, and improve therapeutic responses in liver cancer.

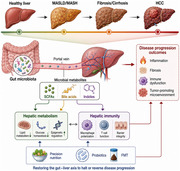

**Abbreviations**
AhR: aryl hydrocarbon receptor
ALD: alcohol‐associated liver disease
BA: bile acid
CAID: cirrhosis‐associated immune dysfunction
CDR: cirrhosis dysbiosis ratio
CDCA: chenodeoxycholic acid
DCA: deoxycholic acid
ECM: extracellular matrix
FIB‐4: fibrosis‐4 index
FXR: farnesoid X receptor
GDCA: glycine‐conjugated deoxycholic acid
GPR/FFAR: G‐protein‐coupled receptor/free fatty acid receptor
HBV: hepatitis B virus
HCC: hepatocellular carcinoma
HCV: hepatitis C virus
HDAC: histone deacetylase
HFD: high‐fat diet
HSC: hepatic stellate cell
ICI: immune checkpoint inhibitor
LCA: lithocholic acid
LPS: lipopolysaccharide
LSEC: liver sinusoidal endothelial cell
MASLD: metabolic dysfunction‐associated steatotic liver disease
MASH: metabolic dysfunction‐associated steatohepatitis
MDSC: myeloid‐derived suppressor cell
NES: normalized enrichment score
NF‐kB: nuclear factor‐kappa B
NFS: NAFLD fibrosis score
NKT: natural killer T cell
PDI: patient dysbiosis index
PXR: pregnane X receptor
ROS: reactive oxygen species
SASP: senescence‐associated secretory phenotype
SCFA: short‐chain fatty acid
TAM: tumor‐associated macrophage
TCDCA: taurochenodeoxycholic acid
TGR5/GPBAR1: G‐protein‐coupled bile acid receptor 1
TLCA: taurine‐conjugated lithocholic acid
TLR: Toll‐like receptor
TME: tumor microenvironment
Treg: regulatory T cell
UDCA: ursodeoxycholic acid
VDR: vitamin D receptor

## 1 Introduction

Chronic liver disease represents one of the most pressing global health burdens, often culminating in hepatocellular carcinoma (HCC), the predominant form of primary liver cancer. Liver cancer currently ranks as the sixth most common malignancy and the third leading cause of cancer‐related death worldwide [1, 2, 3]. HCC accounts for approximately 90% of primary liver cancers and, according to GLOBOCAN estimates, was responsible for roughly 865 000 new cases and 758 000 deaths in 2022 [2]. These sobering statistics underscore the urgent need to define the biological pathways that drive hepatocarcinogenesis.

Liver cancer arises from long‐term liver injury caused by diverse etiologies, including metabolic dysfunction, alcohol exposure, chronic viral hepatitis, cholestatic or autoimmune liver disease, aflatoxin exposure, and increasingly common mixed etiologies [1, 2, 3]. Therefore, the MASLD‐MASH‐fibrosis/cirrhosis‐HCC sequence should not be interpreted as the only route to HCC. Rather, in this review, it is used as a model system in which the dynamic interactions among metabolism, immunity, microbiota, and diet can be mechanistically resolved. Many mechanisms overlap across ALD‐ and viral hepatitis‐associated HCC, including gut barrier failure, microbial translocation, bile‐acid perturbation, chronic inflammation, immune exhaustion, and fibrotic remodeling; however, their relative contributions and temporal order differ by etiology [4, 5, 6, 7, 8].

For example, alcohol‐associated liver disease (ALD) is strongly shaped by ethanol‐induced dysbiosis, epithelial barrier disruption, endotoxemia, acetaldehyde‐mediated hepatocyte injury, and Kupffer‐cell activation [4]. HBV‐ and HCV‐related HCC, by contrast, involve viral antigen persistence, antiviral immune exhaustion, direct or indirect oncogenic effects, and cirrhosis‐associated immune dysfunction, while still intersecting with the gut‐liver axis through microbial products and bile‐acid metabolism [5, 8, 9, 10]. Accordingly, throughout the manuscript, we distinguish evidence derived from MASLD/MASH cohorts from evidence obtained in cirrhosis, viral hepatitis, ALD, or mixed‐etiology HCC, and we avoid generalizing MASLD‐specific findings to all forms of HCC without qualification.

Within this etiological framework, chronic liver disease can be viewed as a multistage but branching process of metabolic, inflammatory, and fibrotic remodeling. In metabolic liver disease, systemic metabolic dysfunction initiates hepatic steatosis and may drive progression across a spectrum that includes: (a) metabolic dysfunction‐associated steatotic liver disease (MASLD), defined by hepatic steatosis in the context of systemic metabolic dysregulation, such as obesity or type 2 diabetes; (b) metabolic dysfunction‐associated steatohepatitis (MASH), an inflection point characterized by hepatocellular injury and inflammation that promote fibrogenesis; and (c) advanced fibrosis and cirrhosis, which establish a permissive microenvironment for malignant transformation and confer a markedly elevated risk of HCC [11, 12, 13]. Although only a subset of patients progresses to these advanced stages, fibrosis severity remains one of the strongest predictors of future liver‐related complications and malignant progression.

Importantly, disease progression is neither uniform nor inevitable. The transition from steatosis to steatohepatitis, fibrosis, cirrhosis, and carcinoma typically unfolds over years, but patients may follow divergent trajectories. Some remain stable or regress after lifestyle or metabolic intervention, whereas others develop advanced fibrosis without a clinically captured inflammatory stage. Moreover, HCC can arise in non‐cirrhotic livers, particularly in metabolic disease and chronic HBV infection. Progression is therefore better understood as a set of branching trajectories shaped by host genetics, sex, age, ethnicity, metabolic status, alcohol exposure, viral factors, diet, medication exposure, and microbiome composition.

Across these trajectories, each stage of chronic liver disease is accompanied by an evolving immune, metabolic, and microbial environment that shapes carcinogenesis. Insulin resistance, lipotoxicity, mitochondrial stress, and oxidative injury contribute to hepatocyte damage, chronic inflammation, and fibrogenesis. Persistent immune activation may initially suppress nascent tumor cells but eventually promote a tumor‐permissive inflammatory niche. In parallel, perturbations of the gut‐liver axis increase intestinal barrier permeability, promote microbial product translocation, and drive hepatic inflammation and fibrosis, thereby fostering HCC development [14, 15, 16, 17, 18, 19]. Thus, a comprehensive understanding of the dynamic metabolic, immune, and microbiome landscape across chronic liver disease is essential for explaining how chronic injury progresses to cancer.

**FIGURE 1:**
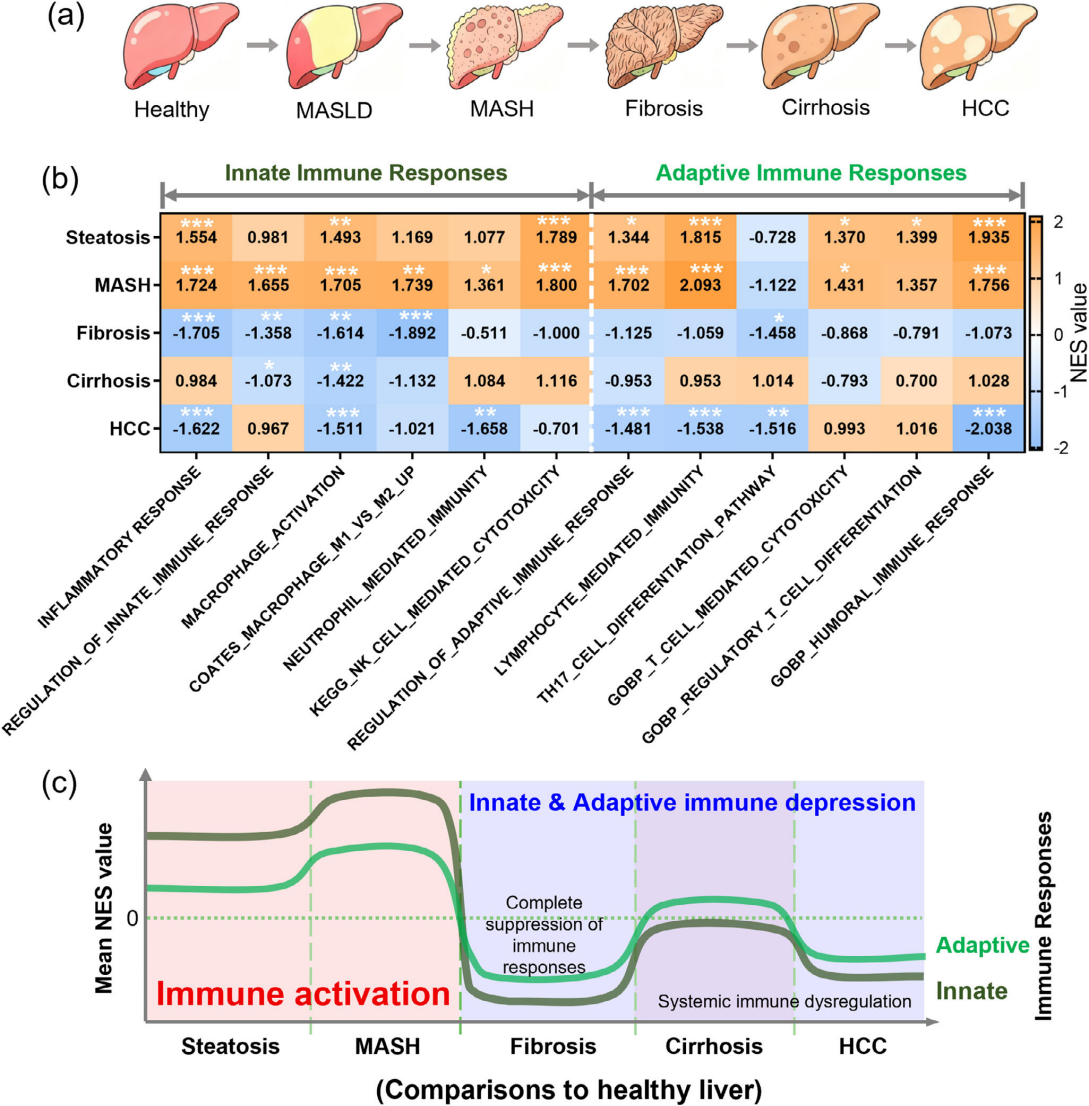
Immune state transitions at the transcriptomic level across the progression from healthy state to MASLD, MASH, fibrosis, cirrhosis, and ultimately HCC. (a) Schematic depiction of liver disease progression from healthy liver to steatosis/early MASLD, MASH, fibrosis, cirrhosis, and ultimately HCC. (b) Heatmap of normalized enrichment scores (NES) derived from gene set enrichment analysis (GSEA) of immune‐related transcriptomic signatures across distinct liver disease stages. Publicly available liver transcriptomic datasets were analyzed by comparing each disease group with its matched control group within the same dataset (bulk RNA‐seq data). Steatosis/early MASLD and MASH were analyzed in GSE48452 (steatosis/early MASLD, *n* = 8; MASH, *n* = 17; healthy controls, *n* = 12), fibrosis and cirrhosis in GSE139602 (fibrosis, *n* = 5; cirrhosis, *n* = 12; controls, *n* = 6), and HCC in GSE112790 (HCC, *n* = 183; non‐cancer liver controls, *n* = 15). Immune pathway gene sets were obtained from MSigDB and the curated gene set collection listed in Excel S1, and enrichment was calculated using GSEA v4.3.2. Positive NES values indicate pathway enrichment in disease relative to control, whereas negative NES values indicate pathway suppression. Significance is indicated by asterisks (^*^, *p* < 0.05; ^**^, *p* < 0.01; ^***^, *p* < 0.001). Since the datasets differ in platform, cohort characteristics, and tissue context, cross‐stage patterns were interpreted cautiously and not treated as direct longitudinal measurements. (c) Conceptual summary of immune‐state transitions across disease progression, based on the mean NES values of innate and adaptive immune pathways shown in panel (b). This summary illustrates a general pattern of immune activation in steatosis/early MASLD and MASH, profound suppression during fibrosis, and progressive immune dysregulation in cirrhosis and HCC. This panel is intended as an integrative visualization of pathway‐level trends rather than a quantitative longitudinal model.

In this review, we examine the sequential and branching transitions from MASLD to MASH, fibrosis, cirrhosis, and ultimately HCC (Figure 1a). We focus on how alterations in the gut microbiota, liver metabolism, and immune responses, and dietary influences converge to shape disease progression. Because this field often relies on heterogeneous patient cohorts and cross‐sectional datasets, we treat transcriptomic and multi‐omic data as snapshots of disease states rather than definitive evidence of a single temporal sequence. This distinction is especially important because cirrhosis and HCC datasets frequently include heterogeneous etiologies, treatments, and tissue sources that may not directly represent progression from MASLD/MASH in the same individuals. By integrating these perspectives, we aim to highlight key pathogenic mechanisms and provide a framework for preventive and therapeutic strategies against HCC.

## 2 Immune Landscape Variation in Liver Disease Progression

### 2.1 Initiation of Inflammation: From Healthy Liver to MASLD

Progression from a healthy liver to chronic liver disease involves a dynamic remodeling of the hepatic immune landscape. In the healthy liver, Kupffer cells, natural killer (NK) cells, T cell subsets, dendritic cells, and other resident or recruited immune populations maintain immune tolerance while preserving antimicrobial and antitumor surveillance [20].

This homeostatic balance is disrupted when excess lipids accumulate in hepatocytes [21, 22]. Hepatic steatosis associated with obesity, type 2 diabetes, hypertension, dyslipidemia, or related cardiometabolic risk factors is now classified as metabolic dysfunction‐associated steatotic liver disease (MASLD). Lipid overload activates stress pathways in hepatocytes and stimulates innate immune responses involving Kupffer cells, recruited monocyte‐derived macrophages, neutrophils, and innate lymphoid populations [23, 24, 25]. Dysfunctional adipose tissue further amplifies this response by releasing nonesterified fatty acids and inflammatory mediators, including TNF‐α, TGF‐β, IL‐1, and IL‐6, thereby sustaining oxidative stress and inflammatory priming within the liver [23, 26, 27].

Consistent with this concept, pathway‐level analyses of steatotic liver samples compared with healthy controls showed increased normalized enrichment scores (NES) for multiple innate and adaptive immune programs (Figure 1b). These transcriptomic patterns should be interpreted as hypothesis‐generating signatures from cross‐sectional cohorts rather than as proof of a deterministic temporal sequence. Nevertheless, they support the view that immune activation is already present at the early MASLD/steatosis stage.

### 2.2 Immune Activation Peaks at MASH

The transition from simple steatosis to metabolic dysfunction‐associated steatohepatitis (MASH) is driven by the convergence of metabolic stress, hepatocyte injury, and inflammatory amplification [11, 13]. Impaired insulin responsiveness, de novo lipogenesis, mitochondrial dysfunction, and lipotoxic lipid species promote oxidative stress and hepatocellular ballooning [23, 27, 28]. Injured hepatocytes release damage‐associated molecular patterns that activate Kupffer cells and recruit inflammatory monocytes, neutrophils, and lymphoid cells.

At this stage, monocyte‐derived macrophages frequently acquire pro‐inflammatory and pro‐fibrotic phenotypes, while neutrophils and other innate immune cells produce reactive oxygen species, proteases, and cytokines that further injure hepatocytes and activate hepatic stellate cells (HSCs) [13, 20, 27, 29]. Adaptive immune cells, including CD4^+^ and CD8^+^ T cells, regulatory T cells, γδ T cells, and B cell subsets, also contribute to the inflammatory and fibrogenic milieu [14, 20]. Thus, MASH represents a pathogenic inflection point at which metabolic dysfunction becomes coupled to sustained immune activation and early matrix remodeling.

NES‐based immune landscape phenotyping (RNA‐seq data) showed stronger activation of immune pathways in MASH than in steatosis, particularly for innate immune programs (Figure 1b). Because these analyses are derived from heterogeneous public datasets, the results should be presented as convergent pathway tendencies and should be validated in independent cohorts and cell‐type‐resolved datasets.

### 2.3 Fibrosis: Immune Remodeling and Emerging Immunosuppressive Signatures

As MASH progresses, chronic hepatocyte damage and unresolved inflammation trigger an aberrant wound‐healing response that drives extracellular matrix (ECM) deposition and liver fibrosis [30, 31]. HSCs are central mediators of this process: after activation by inflammatory, metabolic, and mechanical cues, they transition from quiescent vitamin A‐storing cells into myofibroblast‐like cells that secrete collagen and other ECM components [32, 33]. Macrophages, neutrophils, damaged hepatocytes, and liver sinusoidal endothelial cells (LSECs) all contribute to this fibrogenic niche through TGF‐β, ROS, lipid peroxidation products, hypoxia‐related signals, and vascular capillarization [34, 35, 36, 37, 38, 39, 40, 41, 42].

Fibrosis should not be described as a stage of complete immune suppression. Rather, it is characterized by coexisting inflammatory, fibrogenic, reparative, and immunoregulatory programs [19, 43]. In the analyzed fibrosis/early chronic liver disease samples, several immune‐related gene sets showed significantly reduced NES values compared with controls (Figure 1b), suggesting attenuation of selected immune pathways or emergence of immunoregulatory features. However, active innate immune signaling and macrophage‐HSC crosstalk remain essential drivers of matrix deposition. We therefore interpret the transcriptomic signal as evidence of immune remodeling and emerging dysfunction, not as a global absence of immune activity. This distinction is clinically important. Fibrosis represents a potentially reversible checkpoint in chronic liver disease progression, and interventions that target metabolic stress, macrophage‐HSC crosstalk, endothelial capillarization, or microbiota‐derived inflammatory cues may prevent transition to cirrhosis and reduce the risk of HCC.

### 2.4 Immune Dysregulation in Cirrhosis

Progressive fibrosis can culminate in cirrhosis, a late‐stage condition characterized by distorted hepatic architecture, regenerative nodules, portal hypertension, and impaired liver function. Cirrhosis is accompanied by Cirrhosis‐Associated Immune Dysfunction (CAID) [44, 45], a paradoxical state in which persistent systemic inflammation coexists with immunodeficiency, increasing susceptibility to infection, decompensation, and malignant transformation [46, 47]. The cirrhotic liver shows altered function and spatial organization of macrophages, dendritic cells, lymphocytes, and regulatory populations [44, 45, 48]. In HBV‐related progression, for example, scar‐associated macrophages such as CD9^+^/IL‐18^+^ populations and effector CD4^+^ and CD8^+^ T cell subsets (TNFAIP3^+^, TNF^+^, CD53^+^) expand, whereas some regulatory subsets decline [49, 50]. The resulting immune milieu impairs surveillance and can foster a tumor‐permissive niche in which chronic inflammation coexists with strategies for immune evasion [44, 45, 48]. A shift from adaptive immune dominance toward innate immune and fibroblast‐associated signaling has also been described during cirrhosis progression [45, 51, 52], and MAPK‐related pathways may contribute to this immune remodeling as well [53]. Cirrhosis markedly increases the risk of HCC across etiologies. While lifestyle modification, etiologic treatment, and management of portal‐hypertension complications remain central to care, immune and microbiota‐targeted strategies may eventually complement existing approaches. Given the profound immune dysregulation observed in cirrhosis (Figure 1b,c), immunotherapy (e.g., IL‐12 [54]) and interventions that restore antimicrobial defense without exacerbating inflammation represent an important therapeutic frontier.

### 2.5 Tumor‐Promoting Immune Dysfunction in HCC

Hepatocellular carcinoma (HCC) arises in the setting of cirrhosis, although non‐cirrhotic HCC occurs in selected contexts, especially chronic HBV infection and metabolic liver disease [55, 56]. Malignant transformation is driven by the combined effects of chronic inflammation, fibrotic remodeling, metabolic reprogramming, and impaired immune surveillance [14, 17, 57, 58, 59, 60, 61, 62, 63]. The HCC tumor microenvironment (TME) contains multiple dysfunctional immune populations, including tumor‐associated macrophages (TAMs), exhausted CD8^+^ T cells, immunosuppressive regulatory T cells (Tregs), altered NK cell subsets, and dysfunctional B cells or plasma cell states [58, 61, 63, 64, 65]. Single‐cell analyses have revealed stage‐ and etiology‐dependent immune remodeling during the transition from cirrhosis to HCC. Reported changes include expansion of CD9^+^/IFI6^+^ macrophage states, altered GNLY^+^NK cell populations, increased Tregs, enrichment of LAG3^+^ exhausted CD8^+^ T cells, and depletion of effector CD8^+^ T cells [50]. Tregs in the HCC TME often express immune checkpoints such as PD‐1, LAG3, TIGIT, and CTLA‐4 [66, 67]. B cell compartments are also remodeled, with reports of proliferative, low‐differentiation memory B cell states and plasma‐cell enrichment in HCC compared with cirrhotic or healthy liver [68, 69].

These dysfunctional immune populations, together with their altered spatial distributions, contribute to tumor immune escape [59, 70]. Tumor‐cell metabolic reprogramming further shapes immune function by altering nutrient competition, hypoxia, acidosis, redox state, lipid handling, and amino acid availability [18]. Disorganized tumor vasculature creates high interstitial pressure and physical barriers to immune‐cell infiltration, while hypoxic and acidic niches reinforce macrophage polarization, T‐cell exhaustion, and therapeutic resistance [71, 72, 73]. Thus, HCC should be understood as an immunometabolic ecosystem in which tumor cells, stromal cells, microbiota‐derived signals, and spatially organized immune niches jointly determine disease progression and treatment response.

In summary, progression from healthy liver to HCC is accompanied by a profound but non‐linear reshaping of hepatic immunity. Early metabolic stress and lipid accumulation trigger inflammatory priming in MASLD; MASH couples hepatocyte injury to robust innate and adaptive immune activation; fibrosis combines active inflammatory‐fibrogenic signaling with emerging immunoregulatory features; and cirrhosis and HCC are characterized by severe immune dysfunction that favors tumor‐permissive microenvironments. Normalized enrichment score (NES) analyses across disease states (Figure 1b,c) support these stage‐associated tendencies but should be interpreted cautiously because the underlying datasets are cross‐sectional and etiology‐heterogeneous. Clinically, understanding how immune responses are modulated by metabolic cues and microbiome composition may help identify stage‐specific biomarkers and guide interventions designed to halt disease progression before malignant transformation.

## 3 Metabolic Evolution From Healthy Liver to HCC

### 3.1 Lipid Metabolism Drives Hepatic Steatosis

**FIGURE 2:**
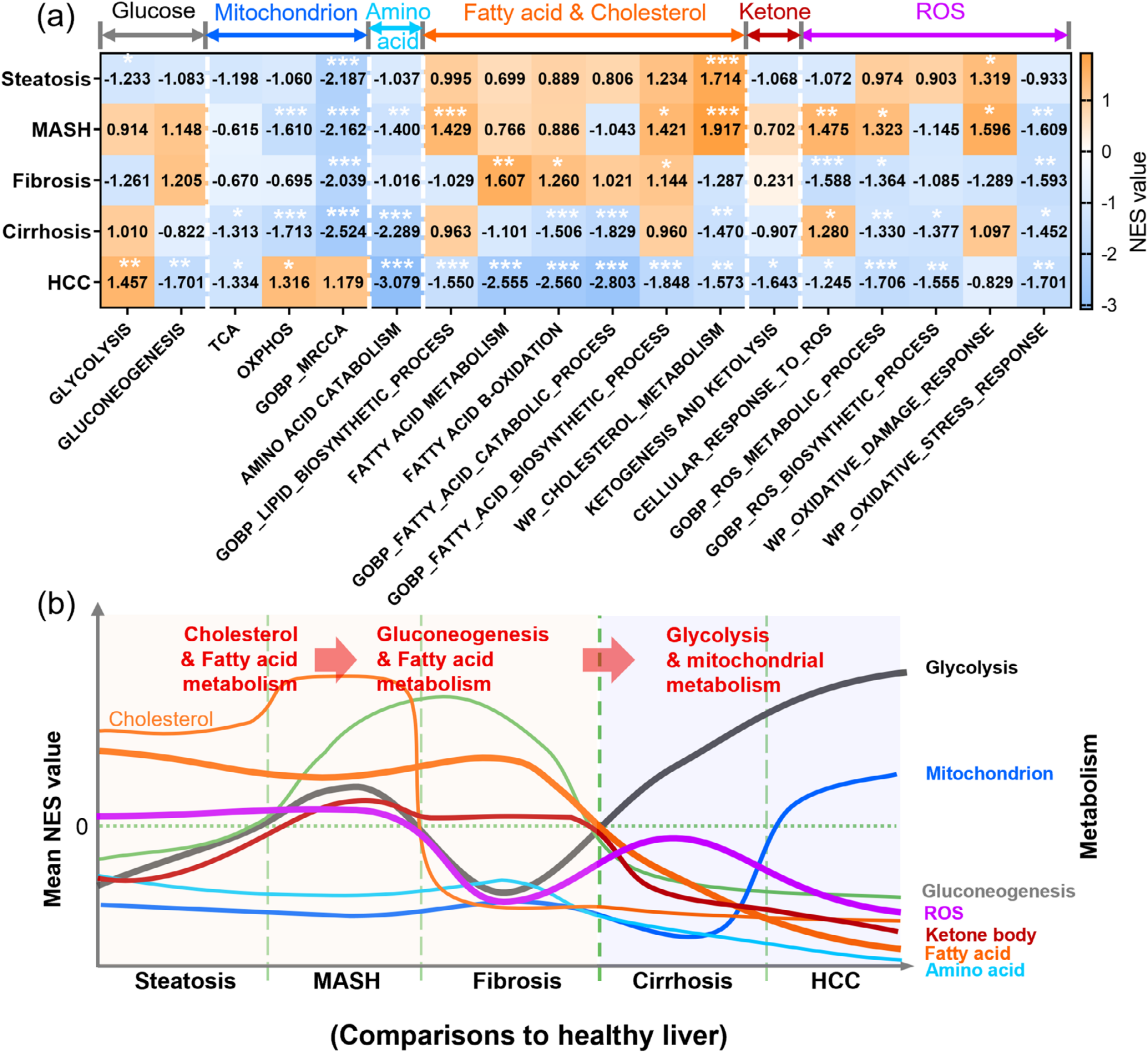
Metabolic transitions during the progression from healthy state to MASLD, MASH, fibrosis, cirrhosis, and eventually HCC. (a) Heatmap of normalized enrichment scores (NES) from RNA‐seq data for metabolism‐related gene sets in steatosis/MASLD, MASH, fibrosis, cirrhosis, and HCC livers. The methodology and datasets used are identical to those used in Figure 1. In early MASLD (steatosis), cholesterol and fatty acid metabolism are greatly enriched, while mitochondrial and amino acid pathways decline, comparing to the healthy controls. Transition to MASH is characterized by enhanced gluconeogenesis, lipotoxicity, and reactive oxygen species (ROS) imbalance, accompanied by mitochondrial dysfunction. With fibrosis and cirrhosis, profound suppression of amino acid metabolism, mitochondrial activity, and oxidative defenses emerges, reflecting systemic metabolic collapse. In HCC, a clear metabolic switch toward glycolysis and mitochondrial reprogramming occurs, consistent with the Warburg effect and supporting tumor proliferation. The number marked is NES value. *, p < 0.05; **, p < 0.01; ***, p<0.001. (b) Schematic pathway dynamics illustrate this sequential shift: lipid metabolism dominates steatosis, gluconeogenesis, lipid biosynthesis, cholesterol metabolism and ROS dysregulation define MASH, metabolic failure marks fibrosis and cirrhosis, and glycolytic reprogramming underlies HCC. Together, this graph highlights a stage‐dependent metabolic trajectory with potential value for biomarker discovery and therapeutic intervention. GEO accession numbers for the above liver conditions are GSE48452, GSE139602, and GSE112790, respectively. Datasets were obtained from GEO (https://www.ncbi.nlm.nih.gov/geo/); gene sets from the Molecular Signatures Database (refer to Excel S2); and GSEA was performed using GSEA v4.3.2 (Broad Institute). MRCCA: Mitochondrial_Respiratory_Chain_Complex_Assembly. TCA: Tricarboxylic acid cycle. OXPHOS: Oxidative Phosphorylation. ROS: Reactive Oxygen Species.

Metabolic reprogramming is central to the pathogenesis of chronic liver disease and HCC [18, 19]. In the healthy liver, lipid and glucose metabolism are tightly coordinated to maintain systemic energy homeostasis. The onset of hepatic steatosis marks a major metabolic shift, with increased fatty acid uptake, de novo lipogenesis, triglyceride storage, and cholesterol handling (Figure 2a) leading to excessive lipid accumulation within hepatocytes [21, 74, 75]. These processes are commonly driven by insulin resistance and cardiometabolic risk factors such as obesity and type 2 diabetes [76]. Excess intrahepatic fat is not merely a passive marker; it increases vulnerability to lipotoxicity, oxidative injury, inflammation, and subsequent disease progression [77].

### 3.2 Metabolic Disorders in MASH

The transition from steatosis to MASH is propelled by escalating metabolic stress [27, 28]. In MASH, dysregulated glucose and lipid metabolism coexist with mitochondrial dysfunction, impaired oxidative defenses, amino acid imbalance, and increased cellular responses to reactive oxygen species (ROS) (Figure 2a). These disturbances promote hepatocyte injury, ballooning, inflammatory signaling, and immune‐cell recruitment [27]. Lipotoxic lipid species further exacerbate ER stress, oxidative stress, and inflammatory cytokine production, thereby linking metabolic overload to the histological features of MASH [27, 78].

### 3.3 Persistent Metabolic Dysregulation in Fibrosis and Cirrhosis

As liver disease advances to fibrosis and cirrhosis, metabolic disruption extends beyond hepatocyte lipid overload to include altered protein, carbohydrate, lipid, bile‐acid, and microbial metabolite metabolism [79, 80, 81]. These changes contribute to insulin resistance, hypermetabolism, and malnutrition in cirrhotic patients despite unchanged or reduced caloric intake [82, 83]. Mitochondrial dysfunction, oxidative stress, hypoxia signaling, and urea‐cycle activity further exacerbate immune and metabolic dysregulation in the diseased liver [19, 84]. Together, these shifts sustain macrophage, lymphoid‐cell, stellate‐cell, and endothelial remodeling, creating a fibrotic and metabolically fragile niche that favors malignant transformation.

### 3.4 Glycolytic Reprogramming in HCC

The development of HCC is closely associated with profound metabolic reprogramming that supports the high anabolic and energetic demands of tumor cells. HCC cells commonly exhibit increased glycolysis (characteristic of the Warburg effect) as well as disruptions in fatty acid catabolism [85, 86] and amino acid metabolism [86, 87, 88, 89, 90]. Consistently, normalized enrichment score (NES) analyses of GEO datasets corroborate these observations (Figure 2a). These metabolic alterations supply the energy and biosynthetic precursors necessary for rapid proliferation and survival. In obesity‐associated HCC, for example, the upregulation of fatty acid‐binding protein 5 (FABP5) promotes tumor growth by increasing lipid uptake and conferring resistance to ferroptosis through the regulation of lipid peroxidation. Inhibiting FABP5 not only induces tumor cell death but also reprograms tumor‐associated macrophages toward a pro‐inflammatory phenotype, thereby enhancing CD8^+^ T cell‐mediated antitumor immunity [86]. Similarly, dysregulation of amino acid metabolism plays a crucial role in HCC progression. Arginine accumulation, driven by impaired urea cycle function and increased uptake, activates RBM39‐mediated oncogenic signaling, forming a self‐reinforcing loop that supports tumor growth [87]. Elevated histidine levels further contribute to immune evasion by suppressing the activity of macrophages and T cells, fostering an immunosuppressive microenvironment [89]. Moreover, HCC cells that acquire resistance to sorafenib undergo metabolic rewiring, shifting from glycolysis to glycerol‐3‐phosphate synthesis. This transition enhances lipid metabolism and antioxidant capacity, enabling the cells to evade ferroptosis [90]. Collectively, these interconnected metabolic changes, involving lipid handling, arginine metabolism, and histidine‐driven immunosuppression, not only sustain tumor cell survival but also reshape the immune microenvironment to favor HCC progression. These insights underscore the potential of therapeutic strategies that simultaneously target tumor metabolism and immune regulation [18, 57].

In summary, the metabolic evolution from a healthy liver to HCC reflects a continuous, stage‐specific reprogramming of energy pathways that not only mirrors but actively drives disease progression. In MASLD, excessive lipid deposition, largely fueled by insulin resistance and de novo lipogenesis, marks the first metabolic inflection point. Progression to MASH involves mitochondrial dysfunction, oxidative stress, and amino acid imbalance, sustaining hepatocyte injury and chronic immune activation. Fibrosis and cirrhosis are characterized by systemic metabolic collapse, encompassing protein, bile acid, and microbial metabolite alterations, leading to hypermetabolism, malnutrition, and immune dysfunction. Ultimately, HCC arises through glycolytic reprogramming, dysregulated lipid handling, and amino acid‐driven oncogenic signaling, collectively fueling tumor proliferation and immune escape. Our validations using NES‐based analyses (Figure 2a,b) corroborate these stage‐dependent shifts, delineating a clear metabolic trajectory from lipid dominance in steatosis to glycolytic dependence in HCC. Clinically, these findings underscore that metabolic alterations are not secondary phenomena but central drivers of disease, highlighting therapeutic opportunities: lipid and ROS modulation in MASLD/MASH, nutritional‐metabolic support in cirrhosis, and metabolism‐immune co‐targeting in HCC. Understanding these processes is critical, especially in the context of the gut‐liver axis, where microbial metabolites profoundly shape host metabolism and immunity, representing the next frontier in disease interception.

## 4 The Gut‐Liver Axis Orchestrates Liver Disease Progression

The gut microbiota, a vast and complex ecosystem of microorganisms residing primarily in the gastrointestinal tract, plays an indispensable role in maintaining human health and significantly influences the pathophysiology of numerous diseases, including those affecting the liver [91, 92, 93, 94]. The liver and the gut are anatomically and functionally connected through the “gut‐liver axis,” a bidirectional communication pathway that involves the portal circulation, bile duct, and systemic circulation. This axis allows the liver to be constantly exposed to circulating nutrients, microbial components, and metabolites derived from the gut microbiota [95]. Disturbances in the gut microbiota, known as dysbiosis, are increasingly recognized as critical factors in the induction and promotion of liver damage progression, from initial steatosis to advanced stages such as cirrhosis and HCC [96, 97, 98]. This progression is marked by distinct alterations in the composition and function of the gut microbial community, which contribute to the pathogenesis of liver damage through mechanisms such as increased intestinal permeability, bacterial translocation, immune dysregulation, inflammation, and altered metabolite production [99, 100, 101]. Understanding these specific microbial shifts is crucial for developing targeted diagnostic and therapeutic strategies [102].

### 4.1 Gut Microbiota Homeostasis in Healthy Liver Function

The gut microbiota and its metabolites are essential for maintaining liver homeostasis. In a healthy state, a diverse microbial community maintains epithelial barrier integrity, immune tolerance, nutrient processing, and host metabolic regulation [103, 104, 105]. The liver is anatomically positioned to sense this microbial ecosystem: nutrients and microbial products absorbed from the intestine reach the liver through the portal vein, while bile acids synthesized in hepatocytes are secreted into the intestine and reshaped by microbial enzymes. Short‐chain fatty acids (SCFAs), including acetate [106], propionate, and butyrate [107], are major products of microbial fermentation of nondigestible carbohydrates [108, 109, 110, 111, 112]. SCFAs support gut barrier function, provide energy to colonocytes, regulate mucus and tight‐junction programs, and modulate hepatic lipid metabolism [108, 113, 114].

Mechanistically, acetate, propionate, and butyrate signal through FFAR2/GPR43 and FFAR3/GPR41 on enteroendocrine, immune, and myeloid populations, thereby influencing GLP‐1 secretion, neutrophil recruitment, macrophage cytokine tone, and systemic insulin sensitivity [115]. Butyrate also activates GPR109A and promotes barrier integrity and regulatory immune programs [112, 116]. In addition, butyrate and propionate refluence gene expression through histone deacetylase inhibition and p300‐related histone acetylation/acylation, linking microbial fermentation to host epigenetic regulation [112, 117]. In the gut‐liver axis, these mechanisms can limit portal LPS delivery and dampen Kupffer‐cell NF‐kB activation; however, excessive acetate generation under high‐fructose conditions may provide carbon for hepatic de novo lipogenesis [118] and tumor‐promoting protein O‐GlcNAcylation [119], highlighting the context dependence of SCFA signaling.

Bile acids represent a second major metabolite‐receptor system [9]. Primary bile acids synthesized in hepatocytes are deconjugated and converted by gut microbes into secondary bile acids [120, 121], which signal through FXR, TGR5/GPBAR1, pregnane X receptor (PXR), VDR, and related receptors [122, 123, 124]. Intestinal FXR activation induces FGF15/19, which returns to the liver through portal circulation to suppress CYP7A1‐mediated bile‐acid synthesis and to regulate triglyceride, cholesterol, and glucose metabolism [125]. FXR signaling also helps restrain inflammation by reducing NF‐kB activity and maintaining barrier function. TGR5 signaling in macrophages, cholangiocytes, Kupffer cells, and enteroendocrine cells increases cAMP, suppresses inflammatory cytokine production, enhances GLP‐1 secretion, and may influence energy expenditure [126]. In HCC, bile‐acid signaling becomes bidirectional and context dependent: secondary bile acids such as deoxycholic acid (DCA) and lithocholic acid (LCA) can induce DNA damage, senescence‐associated secretory phenotype, and impaired NKT‐cell recruitment, whereas selected bile‐acid species may enhance CXCL16‐dependent accumulation of CXCR6^+^ NKT cells in liver sinusoids and support antitumor surveillance [127].

Tryptophan‐derived indoles provide a third signaling axis [128]. Indole‐3‐aldehyde, indole‐3‐propionic acid, indole‐3‐acetic acid, and related microbial products activate aryl hydrocarbon receptor (AhR) and/or PXR in intestinal epithelial cells, immune cells, and hepatocytes [129, 130, 131]. AhR activation promotes IL‐22‐dependent mucosal defense, tight‐junction maintenance, and redox control, whereas PXR activation enhances xenobiotic metabolism, epithelial barrier integrity, and anti‐inflammatory transcriptional programs [132]. Loss of indole‐producing bacteria can therefore increase gut permeability, amplify portal endotoxemia, and alter hepatic macrophage and T‐cell responses [128]. These receptor‐specific mechanisms explain how microbiota composition is translated into host metabolic and immune phenotypes during chronic liver disease progression.

**FIGURE 3:**
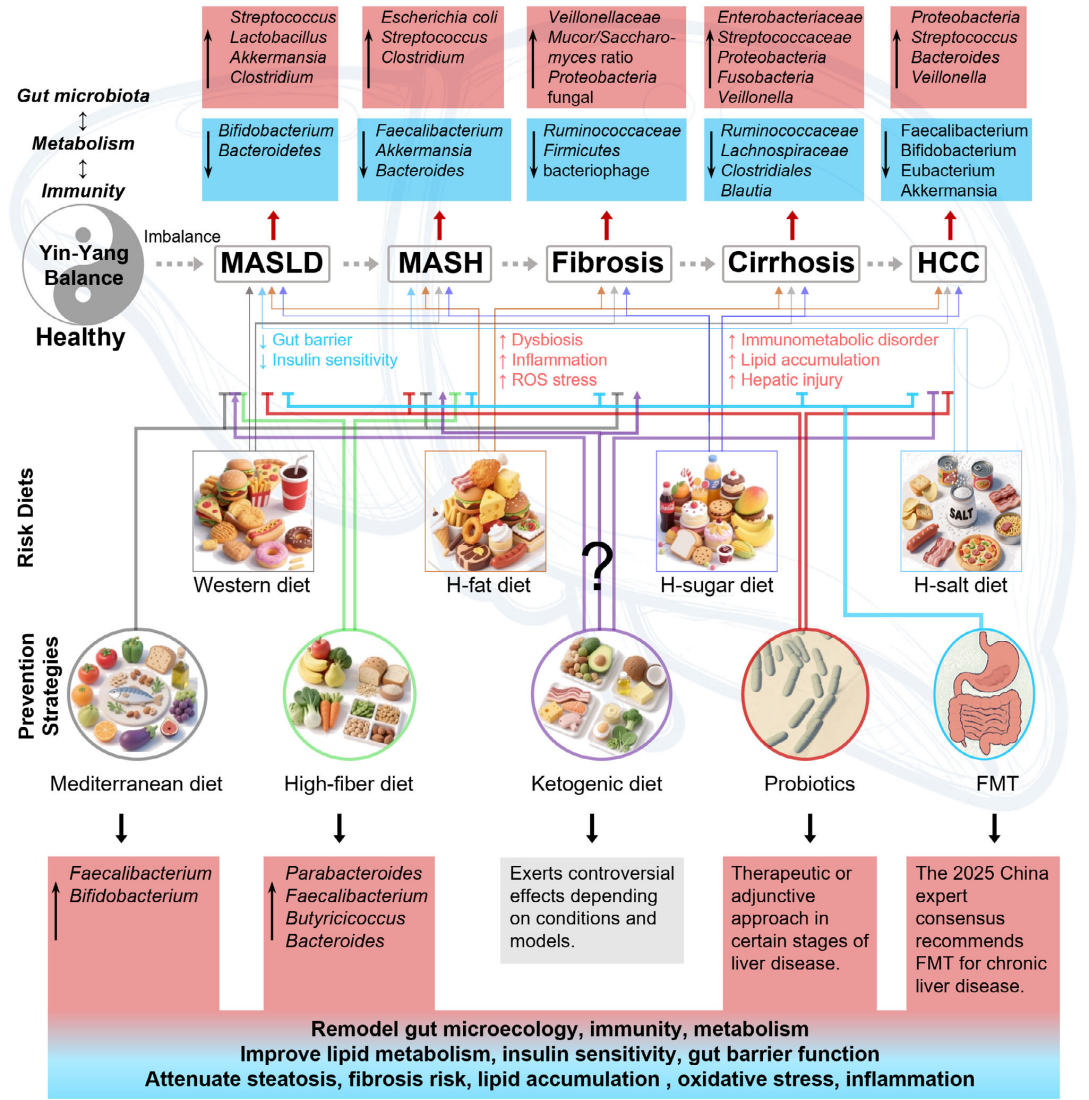
Gut microbiota orchestrates liver disease progression. Risk diets accelerate disease development, while preventive diets and gut microbiota‐targeted remodeling strategies offer promising approaches to prevent or even reverse chronic liver disease. In individuals with a healthy liver, gut microbiota, metabolism, and immune regulation remain in homeostasis, often described as a “Yin–Yang balance”. However, when steatosis or MASLD develops and progresses to advanced stages such as HCC, the gut microbiota undergoes significant alterations and displays distinct features. Over the years, dietary habits have shifted worldwide, with increasing consumption of unhealthy or risk diets that impair liver health. Representative risk diets include the Western diet, high‐fat diet, high‐sugar diet, and high‐salt diet. These patterns disrupt gut barrier function, reduce insulin sensitivity, and induce gut dysbiosis, inflammation, oxidative stress, immunometabolic disorders, lipid accumulation, and hepatic injury. Fortunately, various dietary patterns, nutritional supplementation, and gut microbiota–targeted remodeling strategies can help prevent or even reverse chronic liver disease progression. Representative preventive approaches include the Mediterranean diet, high‐fiber diet, probiotic‐based interventions (or probiotic‐supplemented diets), and fecal microbiota transplantation (FMT). These strategies remodel gut microecology, immune responses, and metabolism by enhancing lipid metabolism, improving insulin sensitivity and gut barrier integrity, and attenuating steatosis, lipid accumulation, oxidative stress, inflammation, and fibrosis risk. Among preventive diets, the ketogenic diet shows controversial effects on liver health, with outcomes varying depending on experimental models and clinical conditions. More in‐depth studies are therefore required to clarify its role in liver disease management. (↕, interaction; →, induction; T, prevention).

Microbial choline metabolism also affects phosphatidylcholine synthesis, lipid export, and steatosis risk [133]. At the community level, the dominant bacterial phyla in healthy adults include *Bacillota* (or *Firmicutes*), *Bacteroidota* (*Bacteroidetes*), *Actinomycetota* (*Actinobacteria*), *Pseudomonadota* (*Proteobacteria*), and *Verrucomicrobiota* [134, 135, 136]. Although the *Firmicutes*/*Bacteroidetes* (F/B) ratio has historically been used as a broad index of microbial balance [137], functional output is more informative than phylum‐level composition. Species such as *Akkermansia muciniphila* and *Faecalibacterium prausnitzii* support intestinal homeostasis and SCFA‐related signaling [113, 114]. Overall, healthy gut‐liver communication depends on balanced microbial metabolism, intact barrier function, regulated bile‐acid cycling, limited Toll‐like receptor activation, and coordinated immune tolerance (Figure 3).

### 4.2 Early Gut Dysbiosis and Functional Shifts in Liver Steatosis

Early hepatic steatosis is frequently accompanied by measurable gut microbiome alterations, even before the development of overt MASH [110, 138, 139]. Reported changes include reduced α diversity, expansion of selected genera such as *Clostridium, Lactobacillus, Streptococcus*, and *Akkermansia* in some cohorts, and depletion of beneficial taxa such as *Bifidobacterium* [139, 140, 141, 142, 143]. Patients with obesity‐associated steatosis often show reduced *Bacteroidetes*‐related taxa, although taxonomic signatures vary by geography, diet, medication exposure, obesity status, and sequencing platform [141, 144].

These microbial changes can promote steatosis through barrier disruption and metabolite remodeling. Increased intestinal permeability permits LPS and other microbe‐associated molecular patterns to reach the liver via the portal vein [100, 145]. LPS activates TLR4‐MYD88‐NF‐κB signaling in Kupffer cells and hepatocytes, increasing TNF‐α and IL‐6 production and reinforcing hepatic lipid accumulation and inflammatory priming [25, 95, 97, 146, 147, 148]. Reduced SCFA availability may further weaken epithelial tight junctions and impair anti‐inflammatory signaling, whereas altered bile‐acid transformation can modify FXR‐FGF15/19 feedback and hepatic cholesterol‐to‐bile‐acid flux [139, 149, 150, 151].

Importantly, early dysbiosis can coordinate with host gene‐expression changes that favor lipid accumulation. Loss of SCFA‐producing taxa and enrichment of endotoxin‐producing pathobionts can increase hepatic expression of inflammatory and lipogenic programs, including pathways related to fatty‐acid uptake, de novo lipogenesis, cholesterol handling, oxidative stress, and cytokine signaling [95, 97, 149, 152, 153]. Thus, microbiome disruption in steatosis should not be viewed as merely correlative; it may reinforce the hepatic transcriptomic shift toward lipid storage and inflammatory susceptibility.

### 4.3 Gut Dysbiosis and Functional Disruptions in MASH

As steatosis progresses to MASH, gut dysbiosis becomes more pronounced and more functionally disruptive [27, 139, 154].MASH is associated with reduced microbial richness, impaired barrier function, endotoxemia, and hepatic inflammation. Several studies report depletion of beneficial or barrier‐supporting taxa such as *Akkermansia muciniphila* [113, 155], *Faecalibacterium prausnitzii* [156], and *Bacteroides uniformis* [155], together with enrichment of pathobionts including *Streptococcus parasanguinis/salivarius*, Escherichia coli, and *Clostridium bolteae* [113, 155, 156].

Functionally, MASH‐associated microbiomes show reduced production of protective metabolites such as 3‐succinylated cholic acid (3‐sucCA), a *Bacteroides uniformis*‐derived metabolite with reported hepatoprotective effects [155]. Altered bile‐acid profiles, including reduced fecal glycine‐conjugated DCA (GDCA), chenodeoxycholic acid (CDCA), and taurine‐conjugated lithocholic acid (TLCA), reflect disrupted microbial bile acid metabolism [156]. Serum bile‐acid alterations, including increases in FXR‐antagonistic DCA in some cohorts, may further impair FXR/TGR5 signaling, immune regulation, and hepatocellular stress responses [157]. MASH microbiome also shows shifts toward stress‐response pathways and away from protein synthesis. These microbial functional features have shown diagnostic potential in multi‐omic models and may offer targets for microbiome‐directed diagnostics and therapy [156].

### 4.4 Multi‐Domain Gut Dysbiosis Defines Liver Fibrosis Progression

Liver fibrosis, a pivotal stage in chronic liver disease, is characterized by the proliferation and deposition of extracellular matrix in liver tissues, often as a result of chronic inflammation [39, 41, 43]. Gut dysbiosis is further altered at this stage, even preceding cirrhosis [97, 110, 158, 159, 160]. Multi‐domain dysbiosis characterizes hepatic fibrosis progression, with reduced microbial diversity universally observed across bacterial, fungal, and viral communities. In non‐obese MASLD patients, advanced fibrosis exhibits bacterial signatures including *Veillonellaceae* enrichment (linked to bile acid disturbances), *Ruminococcaceae* depletion, and elevated fecal propionate‐patterns absent in obese cohorts [110]. Fungal microbiota shifts are particularly prominent in non‐obese individuals, with advanced fibrosis showing increased log‐ratio for *Mucor sp./Saccharomyces cerevisiae (S. cerevisiae)* and elevated systemic anti‐*C. albicans* IgG, indicating immune activation [158]. Virome analysis reveals decreased viral diversity and reduced bacteriophage proportions in advanced fibrosis, with combined viral diversity and clinical metrics yielding high diagnostic accuracy (AUC 0.88 for fibrosis ≥F2) [159]. Metagenomic signatures further identify key taxa (e.g., increased *Proteobacteria*, decreased *Firmicutes*) and dysregulated pathways (e.g., carbon metabolism and detoxification) that distinguish advanced fibrosis from mild disease (AUC 0.936) [160]. Critically, therapeutic modulation via *Ruminococcus faecis* (bacteria) or amphotericin B (antifungal) attenuates fibrosis in murine models, confirming causal roles [110, 158]. Thus, consensus findings demonstrate that fibrosis‐specific dysbiosis involves: (1) bacterial functional shifts (bile acid/SCFA metabolism), (2) fungal pathobiont expansion with immune response, and (3) virome diversity loss, collectively providing non‐invasive diagnostic biomarkers and actionable therapeutic targets for hepatic fibrosis, particularly in non‐obese cohorts.

Additionally, an inverse correlation has been observed between levels of short‐chain fatty acids and prognostic indices of liver disease, such as Non‐alcoholic Fatty Liver Disease Fibrosis Score (NFS) and Fibrosis‐4 (FIB‐4) index, as well as elastography values, indicating that a decrease in SCFA content can be a prognostic marker for liver disease progression [161, 162]. Interestingly, while SCFAs are generally hepatoprotective, their role is context‐dependent. For example, acetate overproduction, particularly via microbial fermentation of dietary fructose by *Bacteroides* and *Lactobacillus*, can contribute to hepatic lipogenesis and potentially exacerbate liver pathology [118].

### 4.5 Progressive Gut Dysbiosis and Functional Shifts in Cirrhosis

Cirrhosis represents the end stage of various chronic liver diseases and is closely associated with significant alterations in the gut microbiota. It promotes progressive gut dysbiosis, as reflected by a declining Cirrhosis Dysbiosis Ratio (CDR), from 2.05 in healthy individuals to 0.32 in hospitalized patients (*p* < 0.0001) [163]. This decline corresponds to a marked depletion of beneficial commensal taxa such as *Lachnospiraceae, Ruminococcaceae*, and *Clostridiales XIV*, alongside a significant enrichment of pathogenic taxa including *Proteobacteria, Fusobacteria*, *Streptococcaceae*, and *Enterobacteriaceae*, compared to healthy controls [163, 164, 165]. One proposed mechanism underlying this shift is impaired bile acid metabolism, characterized by a reduced secondary‐to‐primary bile acid ratio (*p* < 0.05). This is likely due to the loss of 7α‐dehydroxylating bacteria such as *Ruminococcaceae* and *Blautia* [166].

Furthermore, reduced SCFA production compromises gut barrier integrity [163], while enhanced ammonia synthesis, driven by enriched nitrate metabolism pathways, and elevated GABA production have been linked to hepatic encephalopathy [164]. Notably, many of the enriched species, such as *Veillonella atypica* and *Streptococcus salivarius*, are of oral origin [164], highlighting translocation from extraintestinal sites. Together, these factors perpetuate a vicious cycle of bacterial translocation and systemic inflammation.

Clinically, microbial signatures offer non‐invasive diagnostic potential. A validated 15‐gene biomarker panel enables accurate identification of dysbiosis, while both the CDR and the Patient Dysbiosis Index (PDI) have been shown to correlate with disease severity [163, 164]. Beyond diagnostics, these insights also highlight potential therapeutic targets aimed at restoring bile acid and SCFA balance and interrupting gut‐liver pathophysiology.

### 4.6 Gut Dysbiosis and Functional Reprogramming in HCC

HCC is a late and clinically devastating outcome of chronic liver disease and reflects long‐standing disruption of the gut‐liver axis. HCC‐associated microbiomes are often characterized by depletion of beneficial SCFA producers such as *Faecalibacterium*, *Eubacterium*, *Bifidobacterium*, reduced mucosal protectors such as *Akkermansia*, and enrichment of *Proteobacteria*, *Streptococcus spp*., *Veillonella* [139, 167, 168], and enrichment of selected opportunistic genera like *Veillonella parvula* and *Bacteroides spp*. (e.g., *B. caecimuris, B. xylanisolvens* [167, 169]). Taxonomic findings are not fully consistent across studies, partly because of differences in etiology, geography, cirrhosis status, treatment exposure, and sequencing methods [167, 168, 169, 170, 171]. For example, *Clostridium* has been reported as enriched in some HCC cohorts but depleted in others [167, 168]. Therefore, functional interpretation is often more robust than taxonomic generalization.

Functionally, HCC‐associated dysbiosis can promote carcinogenesis through barrier disruption, chronic inflammation, altered SCFA signaling, and bile‐acid remodeling. Reduced α diversity and depletion of butyrate‐producing bacteria may compromise epithelial integrity, while aberrant SCFA‐related pathways may contribute to regulatory T‐cell expansion and CD8^+^ T cell suppression in selected tumor contexts [139, 167, 168, 171]. Bile‐acid dysregulation is particularly important. Conjugated primary bile acids such as taurochenodeoxycholic acid (TCDCA) can impair CD8^+^ T cell function through oxidative stress [172], whereas secondary bile acids such as lithocholic acid (LCA) can reduce NKT cell recruitment and favor immune evasion [127]. Conversely, some bile acid species, including chenodeoxycholic acid (CDCA) and ursodeoxycholic acid (UDCA) in specific contexts, may support antitumor immunity or improve therapeutic response [127, 172, 173, 174, 175, 176, 177, 178]. Thus, the HCC gut‐liver axis is best understood as a context‐dependent network in which microbial metabolites, intestinal barrier function, hepatic inflammation, and immune surveillance jointly shape tumor progression.

## 5 Gut Microbiota‐Metabolism‐Immunity Interactions in Liver Disease

### 5.1 Microbial Metabolite‐Driven Immune Programming

The gut‐liver axis is a bidirectional communication network in which gut microbiota‐derived metabolites serve as molecular signals that modulate hepatic immunity through diverse and sometimes opposing mechanisms. SCFAs illustrate this duality. Butyrate can promote anti‐inflammatory IL‐22 production in innate lymphoid cells (ILCs) via GPR41 signaling and histone deacetylase (HDAC) inhibition [111]. However, in selected MASLD‐HCC contexts, butyrate‐associated pathways may also foster immunosuppression by expanding Tregs and reducing CD8^+^ T cell activity [167]. This balance may also involve SCFA‐mediated epigenetic remodeling of Kupffer cells and hepatic dendritic cells, thereby influencing antigen presentation and T cell priming [179]. Acetate also has context‐dependent effects, with *Bifidobacterium pseudolongum*‐derived acetate suppressing HCC via GPR43‐mediated inhibition of IL‐6/JAK1/STAT3 signaling [180], while high‐fructose diet‐induced acetate promotes tumor progression through O‐GlcNAcylation of eEF1A1 [119]. Similarly, propionate can promote anti‐inflammatory macrophage polarization via PPARγ activation [181], but may exacerbate fibrosis by enhancing HSC activation in specific settings [182].

Bile acid metabolism is equally influential. Secondary bile acids like 3‐oxoLCA suppress TH17 differentiation by directly inhibiting RORγt, whereas isoalloLCA promotes Foxp3^+^ Treg development through mitochondrial ROS generation [121]. Tauroursodeoxycholic acid (TUDCA) mitigates ER stress‐induced inflammasome activation in hepatocytes, emphasizing the metabolic‐immune interplay beyond adaptive lymphocytes [183]. Gut dysbiosis can also promote liver cancer initiation through reduced tryptophan metabolites and increased SREBP2‐driven cholesterol synthesis, effects that may be reversible by selected probiotics like *Lactobacillus reuteri* [184]. Clinically, metabolite‐driven immune programming is relevant because fecal microbiota signatures correlate with immunotherapy responses in HCC [185, 186]. Integrating metabolomic and microbiome profiling may therefore help predict immune checkpoint inhibitor (ICI) outcomes and guide rational combinations.

### 5.2 Barrier Dysfunction and Immune‐Metabolic Crosstalk in Liver Injury

Disruption of intestinal homeostasis triggers a cascade of hepatic injury through multiple interconnected mechanisms. In cirrhosis, microbial translocation allows gut‐derived bacteria to activate tonic interferon signaling in hepatic myeloid cells, leading to IL10‐mediated immunosuppression and impaired bacterial clearance [187]. Loss of gut microbial diversity and enrichment of pathobionts such as *Enterococcus faecalis* exacerbate this process, as cytolysin directly damages hepatocytes [188]. Gut vascular barrier (GVB) breakdown further amplifies injury, with increased *Proteobacteria* abundance promoting mucosal inflammation and permeability [189]. Inflammasome dysregulation further contributes to hepatic pathology, as NLRP6 deficiency depletes the mucin‐degrading bacterium *Akkermansia muciniphila*, promoting hepatic monocytic myeloid‐derived suppressor cell (mMDSC) accumulation and suppressing CD8^+^ T cell function via TLR4 signaling [171]. Lipoteichoic acid (LTA), a cell wall component of Gram‐positive bacteria, triggers IL‐33 and IL‐1β release from senescent hepatic stellate cells (HSCs), thereby activating Tregs and facilitating HCC progression [190]. In parallel, metabolite toxicity exacerbates liver injury through multiple pathways: uric acid activates the NLRP3 inflammasome independently of ROS/AMPK pathways [191]; palmitate drives insulin resistance through NLRP3‐dependent IL‐1β secretion [192]; and deoxycholic acid (DCA) induces HSC senescence and subsequent senescence‐associated secretory phenotype (SASP)‐mediated hepatocarcinogenesis [156]. Emerging evidence suggests that gut‐derived endotoxemia not only promotes Kupffer cell activation [193] but also reprograms hepatic lipid metabolism [194], shifting toward triglyceride accumulation and impaired β‐oxidation, thus linking barrier failure directly to metabolic derangements.

### 5.3 Microbiome‐Driven Spatial Regulation, Hepatic Zonation, and Spatial Omics

The liver is not immunometabolically uniform. Hepatocytes are organized along the porto‐central axis into zones with distinct oxygen tension, nutrient exposure, Wnt/beta‐catenin activity, xenobiotic metabolism, lipid handling, and bile‐acid synthesis [195, 196]. Periportal hepatocytes are preferentially exposed to gut‐derived nutrients, microbial products, and oxygen‐rich portal blood, whereas pericentral hepatocytes exhibit stronger glycolytic, lipogenic, and xenobiotic programs [195, 196]. This zonation provides a spatial scaffold through which gut‐derived signals can generate regional patterns of steatosis, inflammation, fibrosis, and tumor initiation [36, 197].

Microbial and metabolite gradients may influence hepatic zonation through several mechanisms. First, portal delivery of LPS, peptidoglycan, ethanol, acetate, and other metabolites preferentially stimulates periportal Kupffer cells, LSECs, and hepatocytes, amplifying NF‐kB, interferon, and chemokine signaling [97, 198]. Second, bile‐acid gradients regulate hepatocyte and LSEC gene expression through FXR, TGR5, PXR, and VDR, thereby coupling microbial bile‐acid transformation to zonated cholesterol and lipid metabolism [97, 126]. Third, LSEC‐derived chemokines such as CXCL16 and CXCL9 organize NKT‐cell, CD8^+^ T‐cell, and macrophage positioning in the sinusoid, linking microbial metabolites to spatial immune surveillance. Fourth, hypoxia, capillarization, and extracellular‐matrix remodeling in fibrosis distort sinusoidal flow, potentially redistributing microbial and metabolite exposure across the lobule [36].

Single‐cell RNA sequencing, single‐nucleus multi‐omics, spatial transcriptomics, and spatial proteomics have redefined this field by preserving cell identity and tissue location [6, 199, 200, 201]. Spatially resolved studies of NASH‐HCC and viral HCC show that immunosuppressive neighborhoods are not evenly distributed: MDSCs, TAMs, exhausted PD‐1^+^CD8^+^ T cells, and PD‐L1^+^/ICOS^+^ myeloid cells form multicellular niches that are enriched at tumor margins and peritumoral regions, whereas granzyme B^+^ effector T cells may be depleted within tumor cores [6, 199, 200]. Single‐cell atlases further reveal etiology‐specific immune ecosystems, including MMP9^+^ macrophage/TAM states, exhausted T‐cell programs, early tertiary lymphoid structures, and heterogeneous malignant hepatocyte states [201]. These data support a spatial model in which gut‐derived signals, host metabolic zonation, vascular remodeling, and immune‐cell neighborhoods [199] converge to determine whether injury resolves, progresses to fibrosis, or evolves into immune‐evasive HCC.

Whether microbial gradients directly determine the localization of early HCC nodules remains unresolved. Current evidence supports plausibility rather than proof [97, 202]. Portal delivery of microbial products, zonated bile‐acid signaling, and region‐specific immune surveillance can create spatially biased injury and repair programs, but longitudinal human studies linking stool/portal metabolomics to lobular transcriptomics and early tumor mapping are still lacking. Future studies should integrate paired stool metagenomics, portal/peripheral metabolomics, single‐nucleus multi‐omics, spatial transcriptomics/proteomics, and digital pathology to test whether microbial products predict where steatosis, dysplastic foci, or early HCC nodules emerge [202, 203].

The gut microbiome may also shape hepatic immune‐cell positioning through endothelial signaling. Commensal microbes regulate Kupffer and NKT cell positioning via MYD88‐dependent CXCL9 gradients in liver sinusoidal endothelial cells (LSECs) [198], while microbial bile acid metabolism regulates NKT cell localization by modulating CXCL16 expression in liver sinusoids [127]. The liver receives 70% of its blood supply from the gut via the portal vein, creating a privileged route through which microbial products influence hepatic immunity [103, 139, 189, 204]. This organization becomes disrupted in NASH, where CXCR6^+^CD8^+^ T cells can lose compartmental confinement and acquire auto‐aggressive properties driven by IL‐15/FOXO1 signaling and acetate‐mediated metabolic activation [205]. Emerging spatial and single‐cell studies also suggest etiology‐specific immune disruption in HBV‐related versus non‐viral HCC [206, 207]. Evidence for intratumoral bacterial spatial heterogeneity in HCC remains preliminary [189, 198, 208], but the possibility that microbial niches co‐localize with immunosuppressive myeloid clusters is an important area for future validation [208, 209, 210].

### 5.4 The Gut Microbiota‐Hepatic Epigenome Axis

The gut microbiota can influence the hepatic epigenome through metabolite availability, one‐carbon metabolism, chromatin‐modifying enzymes, and inflammatory signaling. SCFAs are the most direct example. Butyrate and propionate alter histone acetylation by inhibiting HDACs and by generating acyl‐CoA substrates that activate p300/CBP‐dependent histone acylation, thereby influencing enhancer accessibility and inflammatory gene expression [117, 211]. In hepatocytes, stellate cells, and macrophages, such chromatin changes may regulate genes involved in fatty‐acid oxidation, collagen production, cytokine secretion, and antigen presentation [211]. Microbial choline metabolism and folate‐dependent one‐carbon flux can also influence S‐adenosylmethionine availability, DNA methylation, and phosphatidylcholine synthesis, thereby connecting dysbiosis to steatosis and carcinogenesis [212, 213]. Human NAFLD liver methylome studies show that disease‐associated methylation changes occur in genes involved in intermediate metabolism and insulin/IGF signaling and may be partially reversible after bariatric surgery [214]. These findings support the concept that microbiota‐directed interventions may modify not only metabolites and immune tone but also durable hepatic transcriptional memory.

### 5.5 Sexual Dimorphism in the Gut‐Liver‐Immune Axis

Sex is a major modifier of MASLD, fibrosis, and HCC risk [215]. Men generally have higher HCC incidence, while premenopausal estrogen exposure appears protective against metabolic inflammation and fibrogenesis; after menopause, the risk of MASLD progression increases, consistent with loss of estrogen‐mediated metabolic and immune regulation [216]. Mechanistically, estrogen receptor signaling can reduce IL‐6 production, improve insulin sensitivity, support mitochondrial function, and modulate macrophage polarization, whereas androgen receptor signaling can promote hepatocyte proliferation, viral replication, lipogenesis, and tumor initiation in context‐dependent ways [215]. These hormone‐dependent effects intersect with the gut microbiota because microbial beta‐glucuronidase activity regulates enterohepatic estrogen recycling, and sex‐specific microbiota can alter bile‐acid pools, SCFA profiles, and immune‐cell maturation [215, 217, 218].

Sexual dimorphism should therefore be considered when interpreting microbiome and dietary studies [215]. A microbiota‐targeted intervention that increases secondary bile acids, for example, may have different metabolic or immune consequences in males and females because bile‐acid synthesis, FXR/TGR5 signaling, hepatic transporter expression, and inflammatory thresholds are sex dependent [122, 126, 215]. Similarly, diet‐induced HCC models can show male‐biased tumorigenesis through pathways involving p62, c‐Myc, oxidative stress, and sphingolipid metabolism [219, 220]. We therefore recommend that future clinical and preclinical studies stratify analyses by sex, menopausal status, hormone therapy, and etiology, and that figure legends specify the sex composition of analyzed cohorts when available.

### 5.6 Limitations of Preclinical Models and Requirements for Validation

A major limitation of gut‐liver axis research is reliance on dietary mouse models that capture selected aspects of human disease but rarely reproduce the full transition from metabolic dysfunction to advanced fibrosis, cirrhosis, and HCC [221]. Short‐term high‐fat diet models robustly induce obesity, insulin resistance, and steatosis but often produce modest inflammation and limited fibrosis [221]. Methionine‐choline‐deficient diets induce steatohepatitis and fibrosis but cause weight loss and do not model the insulin‐resistant human metabolic state [221]. Western diet plus fructose/sugar‐water models better approximate metabolic injury and can progress to fibrosis and HCC over longer durations [222], but tumor penetrance, immune composition, microbiome structure, and bile‐acid pools remain mouse‐strain‐, facility‐, sex‐, and diet‐dependent. Germ‐free and antibiotic‐treated models are powerful for causality but can exaggerate effects because immune development, bile‐acid pools, and epithelial physiology differ from conventionally colonized humans [95, 97].

Accordingly, microbiota‐targeted therapies should be validated across complementary systems [221, 223]: human cohorts with longitudinal stool and blood sampling; gnotobiotic mice colonized with patient‐derived microbiota; organoids or precision‐cut liver slices exposed to defined metabolites; and cell‐type‐specific genetic models targeting hepatocytes, Kupffer cells, LSECs, stellate cells, cholangiocytes, and T cells. Cell‐type resolution is essential because the same microbial metabolite can have opposing effects depending on the responding cell [153]. For example, DCA may promote stellate‐cell senescence and SASP‐mediated tumorigenesis, while selected bile‐acid species can enhance NKT‐cell immunosurveillance [126, 127]. Similarly, SCFAs may strengthen epithelial barrier integrity, yet, in some tumor contexts, expand Tregs or support cancer‐cell metabolism [179, 211]. This complexity argues against one‐size‐fits‐all microbiota therapy and supports mechanism‐guided, cell‐type‐specific validation before clinical translation.

### 5.7 Therapeutic Targeting of the Gut–Liver–Immune Axis: Current Strategies and Future Directions

Rapid advances in understanding the gut microbiota‐metabolism‐immunity axis have revealed diverse therapeutic opportunities, including probiotics, fecal microbiota transplantation (FMT), metabolite‐targeted interventions, dietary strategies, and rational combinations with immunotherapy [139, 224]. Probiotic strategies have emerged as clinically important modulators of the gut‐liver axis in metabolic liver disease (Figure 3). For example, the commensal Kineothrix alysoides markedly alleviated hepatic steatosis and improved lipid metabolism in murine MASLD by suppressing hepatic PPAR‐γ and CD36–mediated fatty acid uptake [225]. Lactobacillus and Pediococcus strains, including L. bulgaricus, L. casei, L. helveticus, P. pentosaceus KID7, reduced liver fat accumulation and serum transaminases in MASLD/MASH models while enhancing gut barrier integrity and reducing endotoxin translocation [226]. These probiotics also suppressed proinflammatory cytokines such as TNF‐α, IL‐1β, and IL‐6 and improved liver histology in preclinical studies. A high‐potency multistrain probiotic combined with lifestyle intervention showed benefits in MASLD patients, including improved histology and ALT levels, potentially through restoration of microbiota balance and reduced endotoxemia [227].

At the mechanistic level, probiotic bacteria secrete bioactive metabolites. Hexadecanedioic acid and 3‐succinylated cholic acid attenuate steatosis, insulin resistance, and oxidative stress while enhancing intestinal barrier function [155, 228]. Indole acetic acid (IAA) and indole propionic acid (IPA) inhibit macrophage NF‐κB signaling and suppress hepatic inflammation in MASLD [229]. Probiotics can also modulate antitumor immunity. For example, *Clostridium–*derived secondary bile acids promote CXCL16‐mediated recruitment of liver NKT cells for HCC immunosurveillance [127], whereas *Akkermansia muciniphila* may enhance MAFLD‐HCC immunotherapy by restoring barrier function, increasing T‐cell infiltration, and reducing immunosuppressive myeloid cells [106, 230, 231]. Together, these data suggest that probiotic interventions (Table 1) can improve liver disease via barrier enhancement, microbial metabolite remodeling, anti‐inflammatory effects, and immune modulation.

**TABLE 1:** Representative gut microbiota‐targeted interventions via probiotic supplementation or FMT.

Probiotics or FMT	MASLD	MASH	Fibrosis	Cirrhosis	HCC	Comment	Key mechanisms
***Kineothrix alysoides***	+	—	—	—	—	Alleviates hepatic steatosis, improves lipid metabolism, modulates gut microbiota balance, suppresses inflammation, reduces liver injury markers (ALT/AST), decreases hepatic lipid accumulation.	Suppresses hepatic PPAR‐γ and CD36 expression, reducing fatty acid uptake and lipid accumulation.
***Lactobacillus strains*,** ***P. pentosaceus KID7***	+	+	—	—	—	Lactobacillus strains (bulgaricus, casei, helveticus) and Pediococcus pentosaceus KID7 alleviates hepatic steatosis, lowers cholesterol, modulates gut microbiota, enhances gut barrier, reduces liver inflammation, fat deposition and endotoxin translocation, lowers risk of liver fibrosis.	Enhances intestinal barrier integrity, reduces endotoxin translocation, suppresses cytokine production (e.g., TNF‐α, IL‐1β, IL‐6) [226].
***Akkermansia muciniphila***	+	+	—	—	—	Improves lipid metabolism, regeneration, TCA cycle and insulin sensitivity, reduces hepatic steatosis and inflammation, modulates gut microbiota, enhances gut barrier function	Acetate‐derived AMP activates hepatic AMPK/SIRT1/PGC‐1α axis to suppress PUFA synthesis and alleviate ferroptosis [106, 231]
	+	—	—	—	+	Attenuate liver steatosis and enhances the effectiveness of PD1 immunotherapy.	Enhances T cell response by suppressing m‐MDSCs and M2 macrophages [230].
***Clostridium* species**	—	—	—	—	+	*P*romote Production of CXCL16, recruiting natural killer T (NKT) cells to perform antitumor surveillance.	Bile acids‐CXCL16/CXCR6‐INFγ^+^NKT antitumor immunity [127].
***Bacteroides uniformis***	+	—	—	—	—	*H*exadecanedioic acid (HDA) and 3‐Succinylated Cholic Acid (3‐sucCA) reduces hepatic lipid accumulation, insulin resistance, inflammation, oxidative stress and hepatic steatosis, inhibits lipogenesis and ferroptosis, modulates gut microbiota, enhances intestinal barrier integrity.	Inhibits IRE1α‐XBP1s pathway, suppresses ferroptosis via Nrf2/SLC7A11/GPX4 axis activation. 3‐sucCA promotes growth of Akkermansia. ([156], [228])
***Bifidobacterium bifidum***	+	—	—	—	—	*B. bifidum*‐derived IAA and *Clostridium sporogenes*‐derived IPA attenuate hepatic steatosis, reduces liver inflammation, improves lipid metabolism, enhances gut barrier function, modulates gut microbiota.	IPA & IAA inhibit macrophage activation, suppressing the NF‐κB signaling pathway [229].
***Fusarium foetens***	—	+	—	—	—	Metabolite FF‐C1 of gut fungus *F. foetens* reduces hepatic steatosis, inflammation, fibrosis, ceramide levels, and improves insulin sensitivity.	FF‐C1 inhibits intestinal ceramide synthase 6 (CerS6), reduces serum ceramide [328].
**Multistrain** **probiotic**	+	—	—	—	—	In combination with lifestyle modifications, it improves liver histology, alanine transaminase levels, and cytokine profiles in patients.	Restoring gut microbiota balance, reducing endotoxemia, and lowering pro‐inflammatory cytokines [227].
**Fecal microbiota transplantation, FMT**	+	—	—	—	—	Effective for lean NAFLD patients due to their inherent gut microbiota deficiency, whereas obese patients' microbiota damage is primarily lifestyle‐driven and less responsive to this treatment.	Restoring gut microbiota balance, with greater clinical efficacy in lean NAFLD patients compared to obese patients [232].
	+	—	—	—	—	FMT from vegan donors alters gut microbiota, plasma metabolites, and liver DNA methylation in patients, influences multi‐omics pathways and epigenetic regulation in the liver.	Microbial‐derived plasma metabolites induce epigenetic reprogramming of liver DNA methylation [233].
	+	—	—	—	—	NCT04465032: Three consecutive FMT from lean donors did not significantly reduce hepatic steatosis, improve glucose tolerance, or alter liver biochemistry in patients compared to autologous FMT.	High baseline gut microbiota diversity in recipients limited donor microbiota engraftment and prevented meaningful shifts in microbial composition [234].
	—	—	—	+	—	Alleviates portal hypertension in cirrhotic rats by reducing portal pressure and intrahepatic vascular resistance, without altering liver fibrosis or function.	Butyrate‐producing bacteria increases serum butyrate levels to inhibit HDAC3 activity, thereby activating the PI3K/Akt/eNOS signaling pathway [235].

FMT is another emerging strategy for targeting the gut‐liver‐immune axis (Table 1). In MASLD, allogeneic FMT from healthy donors has shown donor‐ and host‐dependent metabolic benefits [232]. Lean‐donor FMT produced greater reductions in hepatic steatosis and improved metabolic parameters than obese‐donor FMT in one study, suggesting that donor composition and host microbiota status influence engraftment. Vegan‐donor FMT altered plasma metabolomic profiles and induced genome‐wide epigenetic changes in liver DNA methylation in MASLD patients [233]. However, another controlled trial (NCT04465032) found that three weekly lean‐donor FMTs did not significantly reduce liver fat or improve metabolic endpoints in MASLD, likely because high recipient microbial diversity limited donor engraftment [234]. In cirrhotic models, FMT improved portal hypertension without affecting fibrosis by enriching butyrate‐producing taxa and increasing host butyrate, which inhibited hepatic HDAC3 and activated the PI3K/Akt/eNOS pathway in sinusoidal endothelial cells, thereby increasing nitric oxide levels and reducing portal pressure [235]. These mixed results emphasize the need to refine donor selection, improve engraftment, and combine FMT with dietary or immunomodulatory strategies. In China, FMT has been recommended in expert consensus guidance for selected chronic liver disease contexts [236] (Figure 3).

Metabolite‐targeted approaches are also promising. Targeting gut microbial metabolites such as DCA or lipoteichoic acid (LTA) may attenuate senescence‐associated secretory phenotype (SASP) signaling and HCC progression [174]. Ursodeoxycholic acid (UDCA) supplementation has been linked to improved immune checkpoint inhibitor (ICI) responsiveness [186], and inhibition of bile acid‐CoA:amino acid N‐acyltransferase (BAAT) may potentiate anti‐PD‐1 therapy [172]. Immune reprogramming strategies provide additional avenues, including IL‐15 blockade to limit CXCR6^+^ T cell cytotoxicity [205] and GPR120 agonists to suppress NLRP3 activation [237].

Dietary interventions represent another attractive but context‐dependent strategy. Ketogenic diet can suppress HCC growth in some models through HMGCS2 upregulation or ALDOB‐related mechanisms [238, 239], whereas high‐fat, high‐fructose diets disrupt the gut‐liver axis by fostering pro‐inflammatory dysbiosis and amplifying endotoxin‐driven hepatic injury [96]. Because dietary responses depend on disease stage, baseline microbiome composition, metabolic status, and tumor context, dietary strategies should be clinically monitored and ideally guided by functional biomarkers. These issues are discussed in detail in the following section.

Beyond microbiota‐directed interventions, drugs, supplements, and active herb ingredients have shown potential to ameliorate liver disease, as summarized in Table S1 and Table S2. Semaglutide, a GLP‐1 receptor agonist, has demonstrated significant efficacy in MASH clinical trials [240, 241]. Other agents, including tirzepatide [242], statins [243, 244], and bempedoic acid [16], may improve steatosis, inflammation, fibrosis, and metabolic dysfunction. Supplements such as vitamin [16, 245], caffeine [246], and glutamate [247] may reduce oxidative stress, restore autophagy, or support liver regeneration. Active herbal compounds, including cordycepin [248], salidroside [249], alnustone [250], berberine [251], and ponicidin [252] (Table S1), have been reported to attenuate lipid accumulation, inflammation, and fibrosis, gut‐liver crosstalk, and, in selected models, HCC progression.

Cellular immunotherapy may also intersect with microbiota‐directed strategies. Our previous clinical trial showed that immune normalization using allogeneic Vδ2^+^γδT cell therapy prolonged survival and improved quality of life in liver cancer patients [105, 253]. Given the close interplays between the gut microbiota and hepatic immunity, a sequential or combination approach that first restores gut microecology and then enhances antitumor cellular immunity is an attractive hypothesis. However, this concept should be tested rigorously in mechanistic models and prospective clinical trials before clinical adoption. Fundamental questions remain: how microbial communities evolve across disease stages, how host genetics and sex shape metabolite responses, and how tissue‐specific immune cells interpret microbial cues. Integrating multi‐omic microbial and metabolic profiling with precision interventions may reveal actionable targets and transform chronic liver disease management.

## 6 Dietary Modulation of Liver Disease: Risks and Therapeutic Opportunities

Building on the above therapeutic insights, it is important to recognize that diet represents one of the most powerful and modifiable determinants of the gut–liver–immune axis. Unlike pharmacological or cellular therapies, dietary exposures continuously shape microbial ecology, metabolite availability, and immune programming throughout the course of liver disease. Thus, understanding how specific dietary patterns drive or mitigate hepatic injury is not only mechanistically informative but also of immediate translational relevance. In this context, we next discuss dietary risk factors that promote disease progression, as well as nutritional interventions with the potential to prevent, attenuate, or even reverse liver pathology.

### 6.1 Risk Dietary Drivers of Liver Disease Progression

The gut microbiota plays a pivotal role in liver disease progression and is strongly influenced by dietary patterns [222, 254, 255, 256, 257, 258]. Unhealthy diets, especially those high in saturated, refined carbohydrates, fructose, and alcohol, promote hepatic lipid accumulation and metabolic stress, thereby contributing to steatosis [254, 259, 260, 261, 262]. As metabolic dysfunction worsens, excess refined carbohydrate intake combined with insufficient dietary fiber can further impair intestinal barrier integrity, allowing inflammatory mediators and microbial products to reach the liver and contribute to MASH [263, 264]. With advancing disease, high intake of heme iron‐rich red or processed meat and alcohol can exacerbate oxidative stress and HSC activation, accelerating fibrogenesis [259, 260, 265, 266, 267]. Foodborne carcinogens such as aflatoxins, particularly when combined with chronic alcohol consumption or viral hepatitis, further increase HCC risk [268, 269, 270].

Representative harmful dietary patterns include the Western diet, high‐fat diet, high‐sugar/fructose diet, high‐salt diet, and context‐dependent ketogenic diet (Figure 3, Table 2). These diets promote liver disease primarily through microbiome disruption, barrier dysfunction, altered bile‐acid and SCFA metabolism, oxidative stress, and immune dysregulation.

**TABLE 2:** Representative diets induce liver diseases.

Diets	MASLD	MASH	Fibrosis	Cirrhosis	HCC	Comment	Key mechanisms
Western diet, WD	+	+	+	+	+	Develops obesity, insulin resistance, hypertriglyceridemia, increases LDL cholesterol in mice. Induces inflammation, dysbiosis, steatohepatitis, liver fibrosis, and liver cancer.	Drives hepatic lipogenesis, ER stress, inflammation, and apoptosis, promoting fibrosis and liver cancer via TGF‐β and collagen networks [255] and gut microbiota imbalances [271].
	+	+	+	—	—	*Blautia producta* produced 2‐OG leads to fatty degeneration and fibrosis.	2‐OG activates HSCs by modulating Mφ through GPR119/TAK1/NF‐κB/TGF‐β1 axis [222].
	+	+	—	—	—	The H_2_S produced by *Vibrio desthiouris* leads to fatty degeneration but the likelihood of fibrosis and liver cancer decreases.	H_2_S activates cDC1 cell autophagy in a c‐kit‐dependent manner, leading to impaired hepatic immune tolerance [256].
WD+JAM‐A^KO^	+	+	+	—	—	Triggers steatosis and fibrotic liver injury in JAM‐A knockout mice	Increased *Veillonellaceae* and *Lactobacillaceae* strains promote the deconjugation of bile acids, and accumulate toxic bile acids [329].
WD+ *ALMS1* ^KO^	+	+	+	+	+	Mice with *ALMS1*‐deficiency (Foz/Foz) fed a Western diet develop cirrhosis and HCC within 24 weeks.	Disrupts the intestinal barrier, increases *Firmicutes*/ *Bacteroidetes* ratio, causes lipopolysaccharide leakage, leads to severe liver inflammation [272].
WD+ CCl_4_	+	+	+	+	+	Exhibits rapid progression of advanced fibrosis and HCC.	Induces the proliferation and activation of HSCs earlier than WD alone. ([203], [273])
High‐fat diet, HFD	+	+	—	—	—	Induces insulin resistance, hyperglycemia, hepatic fat accumulation, and development of MASLD.	Upregulates hepatic lipid uptake gene CD36 and nascent adipose synthesis genes (SREBP1c/ChREBP) [284].
						Maternal HFD with sucralose intake reduces butyrate producers (e.g., *Clostridium XIVa*), worsens hepatic steatosis, disrupts fatty acid biosynthesis and metabolism, and induces gut dysbiosis in offspring.	Reduced butyrate‐GPR43 signaling impairs gut barrier and drives steatosis via the gut‐liver axis [258].
HFD+binge ethanol intake	+	+	+	—	—	Induces DNA damage, oxidative stress, and endogenous apoptosis in hepatocytes, promotes occurrence of obese fatty liver disease.	CXCL1‐recruited neutrophils generate ROS, activating ASK1‐p38 MAPK and ER stress to induce hepatocyte apoptosis [265].
	+	+	—	—	—	Short‐ & long‐term HFD plus acute ethanol binge intake exacerbates neutrophil infiltration, steatohepatitis and damage to the liver.	Elevated FFAs activate ERK/JNK/NF‐κB, upregulating hepatic CXCL1 and then inducing acute liver injury [259].
	+	+	—	—	—	High‐alcohol‐producing *Klebsiella pneumoniae* is over‐fermented in the intestine to produce ethanol, inducing MASLD.	*K. pneumoniae* increases blood alcohol inducing inflammation, mitochondrial dysfunction, hepatic steatosis [260].
HFD+high sucrose	+	+	+	—	—	Induces severe hepatic steatosis, inflammation and fibrosis.	KHK‐C‐driven fructose metabolism induces ATP loss, uric acid accumulation, and oxidative stress‐mediated liver injury [261].
	+	+	+	+	+	The mouse model fed with high‐fat and high‐cholesterol diet (42% fat and 0.1% cholesterol) combined with high‐fructose/glucose drinking (23.1 g/L fructose and 18.9 g/L glucose) simulates the entire process from fatty liver to liver fibrosis and then to liver cancer in human.	High‐fat, high‐cholesterol and fructose/glucose intake drives obesity, metabolic dysfunction, steatohepatitis, and liver cancer. ([259], [261], [285])
HF/CD	+	+	+	+	+	HFD plus high‐cholesterol (HF/CD) induces the development of MASLD and HCC.	Gut microbial shifts under this diet pattern link TCA/IPA imbalance to steatosis, inflammation, fibrosis, and liver cancer. ([172], [285])
HFD+ bacteria	+	+	_	_	—	HFD plus pathogenic gut bacteria *Enterobacteriaceae strains* promote obesity, insulin resistance, and liver fat accumulation.	LPS‐TLR4 signaling induces inflammation, metabolic disorders, and hepatic steatosis [194].
Ketogenic diet, KD	+	+	+	—	—	Induces hepatic steatosis within 3 days, progressing to steatohepatitis and fibrosis after 16 weeks.	IL‐6‐JNK signaling leads to repressed insulin signaling, involving in the pathogenesis of liver diseases [323].
Kethionine‐choline‐deficient diet, MCD	+	+	—	—	—	MCD with high content of polyunsaturated fatty acids results in hepatic lipid peroxidation, expression of pro‐inflammatory genes, and histological inflammation.	Oxidative stress (elevated TBARS) and pro‐inflammatory genes (TNF, COX‐2, iNOS, etc.) drive inflammatory responses [291].
	+	+	—	—	—	Leads to fatty liver.	GCN2‐eIF2α‐CHOP activation mediates ISR, with JNK‐linked lipid accumulation as the primary injury mechanism [293].
	+	+	—	—	—	Induces fatty liver and exacerbates liver injury in db/db mice with diabetes.	FATP4‐mediated fatty acid uptake and impaired VLDL secretion drive steatosis; visceral fat worsens inflammation in db/db mice [292].
MCDDS	+	+	—	—	—	MCD plus dietary sucrose (MCDDS) induces hepatic steatosis, hepatocyte apoptosis, elevated transaminases, lipid peroxidation, and hepatitis.	Sucrose‐induced DNL promotes lipotoxicity and cell death, whereas starch is protective [294].
High‐sugar diet	+	+	—	—	—	High dietary fructose induces MASLD.	Hepatic KHK‐C activity [262] and USP2‐C/EBPα‐11β‐HSD1 axis [287] involve in triggering metabolic stress, transcriptional reprogramming, and liver lipid deposition.
	+	+	—	—	—	Excessive intake of fructose increases the risk of obesity, metabolic fatty liver disease, cardiovascular disease, and mortality.	High fructose overwhelms intestinal metabolism, triggering hepatic lipid accumulation and gut barrier dysfunction [286].
	+	+	+	+	+	Leads to fat degeneration and the development of liver cancer.	F1P‐induced ER stress and endotoxemia activate TNF‐caspase‐2‐SREBP1 axis, promoting liver fat accumulation [254].
High‐salt diet	+	—	—	—	—	High salt diet during the perinatal period induces metabolic fatty liver disease in weaned mice, accompanied by intestinal barrier damage, systemic inflammation, and liver lipid metabolism disorders.	High‐salt diet induces gut dysbiosis, endotoxemia, and FXR‐mediated hepatic lipogenesis [288].
	+	+	—	—	—	High‐salt diet+ antibiotics (penicillin) or alcohol intake exacerbate liver steatosis, leading to gut microbiota imbalance, increased intestinal permeability, and liver mitochondrial dysfunction.	Intestinal dysbiosis disrupts metabolites and gut barrier, promoting hepatic oxidative stress and steatosis [289, 330].
	+	+	—	—	—	Exacerbates the progression of high‐fat diet‐induced steatohepatitis in mice, leading to increased oxidative stress and inflammatory responses.	High salt activates hepatic NADPH oxidase, driving ROS, lipid peroxidation, inflammation, and fibrosis [290].
RPMD	+	—	—	—	—	Red and processed meat diet (RPMD) correlates with insulin resistance and metabolic liver diseases.	Heterocyclic amines promote insulin resistance, [267] drive hepatic lipid accumulation and TGF‐β‐mediated MASH [266].
Soluble Fiber diet	+	+	+	+	+	Long‐term high fermented‐fiber intake alters gut microbiota, enriching Firmicutes and Clostridium, promoting HCC susceptibility	Gut microbiota dysbiosis and secondary bile acids drive TLR4‐mediated liver inflammation and HCC development [263].
	+	+	+	—	—	Feeding inulin caused some mice to develop cholestasis, which subsequently progressed to liver cancer.	Loss of secondary bile acids under vancomycin correlates with reduced HCC despite persistent cholemia [264].
High‐fiber diet	+	+	+	+	+	High intake of dietary fiber and a low intake of total sugars are associated with a lower risk of hepatocellular carcinoma.	High fiber lowers cholesterol by reducing intestinal bile salt absorption, increasing fecal excretion and hepatic synthesis, potentially lowering liver cancer risk [331].

*Note*: ‘+’, induction of liver diseases; ‘‐’, no induction of liver diseases.

The Western diet (WD) is characterized by high consumption of processed foods, refined grains, red and processed meats, sugary drinks, and high‐fat dairy, with low intake of fruits, vegetables, legumes, and whole grains. Animal studies show that WD can promote steatosis, MASH, fibrosis, cirrhosis, and HCC [222, 255, 256, 271]. Mechanistically, WD induces obesity, dyslipidemia, inflammatory cytokine production (TNF‐α, IL‐6), reduced butyrate output, hepatic de novo lipogenesis (FAS/ACC), ER stress (PERK/CHOP), apoptosis (BIM/PUMA), and fibrogenic signaling [255, 271]. Additional mechanisms include HSC activation by 2‐oxoglutarate (2‐OG) through GPR119/TAK1/NF‐κB/TGF‐β1 signaling [222] and impaired immune tolerance caused by H_2_S‐producing gut microbiota, such as *Vibrio desthiouris*, through cDC1 autophagy disruption [256]. WD also accelerates HCC development in Alms1‐deficient mice through gut barrier disruption, dysbiosis, LPS leakage, and intestinal inflammation [272] and can facilitate CCl4‐induced HCC model establishment [202, 273]. Human cohort studies consistently associate WD‐like patterns with liver diseases, HCC risk [274, 275, 276, 277], cardiovascular disease [278], several cancers [257, 278, 279, 280], and even behavioral disorders [281] and increased toxic metal burden [282]. WD‐related injury may be mitigated in selected settings by microbiota interventions, such as *Bifidobacterium bifidum* [229], *Lactococcus lactis* [283]) or antifungal treatments [158].

**FIGURE 4:**
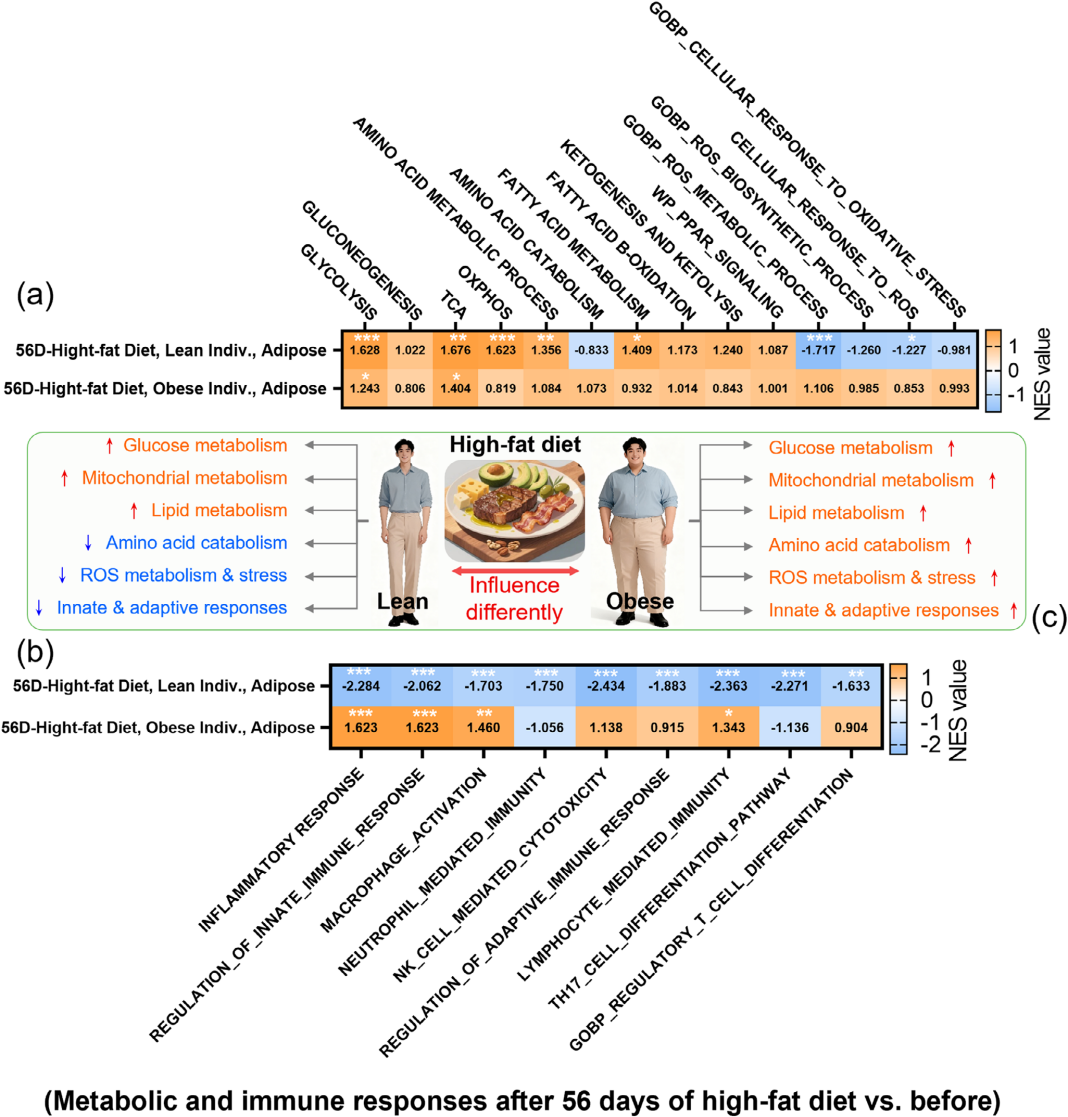
Metabolic and immune phenotype alterations in adipose tissue from lean and obese individuals after 56 days of a high‐fat diet (HFD). Heatmaps show normalized enrichment scores (NES) from RNA‐seq data for (a) metabolism‐ and (b) immune‐related gene sets, and (c) a schematic diagram summarizes immune and metabolic alterations. The number marked (a, b) is NES value. ^*^, *p* < 0.05; ^**^, *p* < 0.01; ^***^, *p* < 0.001. n_lean_ = 6; n_obese_ = 7. Data were obtained from GEO (GSE28005), with gene sets from the Molecular Signatures Database (refer to Excels S1 and S2), and GSEA performed using v4.3.2 (Broad Institute).

A high‐fat diet (HFD), commonly defined as deriving ≥35% of daily calories from fat, is strongly implicated in metabolic liver disease. HFD promotes MASH by reshaping the microbiota, increasing hepatic lipid uptake through CD36, and activating lipogenic transcription factors such as SREBP1c and ChREBP, often in the setting of insulin resistance and hyperglycemia [284]. When combined with ethanol, HFD further exacerbates liver injury [259, 260, 265]. Concurrent consumption of sucrose, cholesterol, or food additives such as sucralose can intensify dyslipidemia, insulin resistance, obesity, and hepatic injury, fibrosis, and carcinogenesis [258, 261, 285]. Transcriptomic analyses of HFD exposure (Figure 4) suggest that immune and metabolic responses differ between lean and obese individuals. In lean individuals, 56 days of HFD led to upregulation of glucose, mitochondrial, and lipid metabolism, coupled with downregulation of amino acid metabolism, ROS metabolism, and stress‐response pathways. Strikingly, gene sets associated with both innate and adaptive immune responses were significantly downregulated at the pathway level. By contrast, obese individuals subjected to the same HFD regimen exhibited broad activation of immune responses along with upregulation of diverse metabolism‐related pathways. These highlight the importance of baseline metabolic and immune context when interpreting dietary effects.

High‐sugar/fructose diets (HS/FD) disrupt intestinal and hepatic metabolism by depleting ATP, increasing uric acid and ROS generation [286], activating lipogenic transcription factors (SREBP‐1c/ChREBP) [262], inhibiting AMPK, and promoting the USP2‐C/EBPα‐11β‐HSD1‐cortisol signaling pathway [287]. Together, these pathways drive lipogenesis and impair fatty acid oxidation. Fructose‐derived metabolites can also damage intestinal barrier integrity, enabling endotoxin translocation and activating hepatic TLR4‐TNF‐SREBP1 signaling [254].

High‐salt diets (HSD) disrupt gut microbiota composition and barrier integrity, increase endotoxin leakage [288], alter bile acid and tryptophan metabolism [289], and activate hepatic lipogenesis pathways [288], promoting oxidative stress through NADPH oxidase activation and Nrf2 inhibition, thereby enhancing hepatic inflammation and eventual liver injury [290].

Other dietary patterns implicated in liver disease progression include the methionine‐choline‐deficient (MCD) diets [291, 292, 293], MCD plus sucrose diets [294], high red and processed meat diets [267], refined grains diets [295], and certain soluble fiber‐rich diets under specific experimental conditions [263] (Table 2). Although their compositions vary, harmful patterns often combine high intake of fat, sugar, salt, alcohol, or processed meat with low intake of fruits, vegetables, whole grains, legumes, nuts, and unsaturated fatty acids. This imbalance disrupts gut‐liver homeostasis and promotes metabolic and inflammatory stress, thereby aggravating liver injury.

Collectively, diets are a central modifier of liver disease risk and progression. Harmful dietary patterns drive hepatic lipid accumulation, inflammation, fibrosis, and carcinogenesis, whereas diets rich in plant‐based foods, fiber, and unsaturated fats generally support metabolic balance and gut‐liver integrity. Understanding these diet‐microbiota‐liver interactions provides a foundation for targeted nutritional strategies in chronic liver disease prevention and management.

### 6.2 Dietary Prevention Strategies for Liver Disease

The gut microbiota exists in a dynamic equilibrium that can be disrupted by harmful dietary patterns but partially restored through targeted nutritional interventions. Clinical and experimental evidence indicate that diet affects liver disease onset and progression from steatosis to HCC. Mediterranean, high‐fiber, and fermented food‐rich diets have shown benefits in MASH and hepatic fibrosis, largely by improving metabolic health, microbiome composition, intestinal barrier integrity, and inflammatory tone [296, 297, 298, 299, 300] (Figure 3, Table 3).

**TABLE 3:** Representative researches of diets protect development and progression of liver diseases.

Diets	MASLD	MASH	Fibrosis	Cirrhosis	HCC	Comment	Key mechanisms
Mediterranean diet	+	+	+	—	—	Reduces liver fat deposition, alleviates hepatic steatosis, lowers risk of liver fibrosis, improves metabolic syndrome, anti‐inflammatory, modulates gut microbiota, inhibits lipid peroxidation, reduces hepatocyte apoptosis, and increases systemic polyphenol levels.	Reduces ceramide synthesis, improves insulin sensitivity and mitochondrial function, lowers hepatic fat accumulation [301], [302, 332].
Mediterranean Diet + Exercise	+	+	+	—	—	Improves hepatic steatosis, alleviates steatohepatitis, promotes fibrosis regression, and is enhanced by increased coffee/nut intake, alcohol avoidance, and physical activity.	Reduces liver fat content (PDFF), normalizes ALT levels, and decreases fibrosis staging via metabolic reprogramming and anti‐inflammatory effects [303].
Low‐carb high‐fat diet (LCHFD);5:2 intermittent fasting	+	+	+	—	+	Reduces liver steatosis and fibrosis, promotes weight loss, lowers LDL cholesterol, enhances metabolic health, attenuates inflammation, enhances antioxidant mechanisms.	Improves insulin sensitivity, reduces hepatic lipid accumulation. Hepatic PPARα and PCK1 are key executors [304, 305].
Low‐fat/high‐carbohydrate diet (LF/HCD)	+	+	—	—	—	Improves hepatic steatosis and insulin sensitivity.	Monounsaturated fatty acids (MUFA) reduce hepatic lipid accumulation [302].
Low carbohydrate diet (LCD); Low fat diet (LFD)	+	—	—	—	—	Reduces liver fat, body weight, improves insulin resistance.	Reduces hepatic de novo lipogenesis and improves insulin sensitivity, decreases liver fat accumulation [306].
Ketogenic diet (KD)	+	—	—	—	+	Reduces hepatic steatosis, improves insulin sensitivity, decreases liver fat content, lowers serum insulin and triglyceride levels, enhances ketogenesis, and reduces markers of liver injury, inhibits HCC.	Lowers insulin levels and hepatic citrate synthase flux, increasing mitochondrial redox state and redirecting fatty acids toward ketogenesis instead of triglyceride synthesis. ([240], [317])
Low protein diet (LPD, 5%kcal)	+	+	—	—	—	Reduces hepatic steatosis, decreases de novo lipogenesis, ameliorates liver injury, and potentially slows disease progression.	Amino acids (e.g., glutamine) contribute to hepatic de novo lipogenesis (DNL) via the TCA cycle and reductive carboxylation [311].
EAT‐Lancet reference diet	+	—	—	—	—	Reduces risk of MASLD, decreases liver steatosis, improves metabolic function, inhibits inflammatory response, and slows fibrosis progression.	Suppresses inflammatory pathways, reduces hepatic fat accumulation [312].
Soluble dietary fiber	+	—	—	—	—	Soluble dietary fiber (20% inulin and oligofructose) alleviates alcoholic liver injury by enriching Bacteroides acidifaciens, reduces hepatic steatosis, inflammation, ammonia levels, oxidative stress, and improves intestinal barrier integrity.	*B. acidifaciens* generates bile acids, activates the intestinal FXR‐FGF15‐OAT signaling axis [298]reducing fatty acid uptake and lipid accumulation Inulin suppress fructose‐related MASLD progression via *B. acidifaciens* [313].
ATI‐free (gluten‐reduced) diet	+	+	—	—	—	Short‐term ATI(Amylase Trypsin Inhibitor)‐free (gluten‐reduced) diet significantly improves liver fat content, insulin resistance, BMI, exhibits anti‐inflammatory and metabolic benefits.	Inactivates intestinal myeloid cells via Toll‐like receptor 4 (TLR4), reduces gut and systemic inflammation [314].
Resistant starch diet	+	—	—	—	—	Reduces intrahepatic triglycerides, improves liver enzymes, decreases systemic inflammation, enhances insulin sensitivity, modulates gut microbiota, lowers LPS and BCAA levels, ameliorates MASLD independently of weight loss.	Reduces productions of LPS and BCAAs via decreases of gut microbiota Bacteroides stercoris [297].
Stearic acid‐enriched diet	+	—	—	—	—	Reduces hepatic steatosis, decreases liver fat accumulation, improves glucose metabolism, alters bile acid profiles, and exhibits anti‐inflammatory and metabolically beneficial effects.	Increased gut Akkermansia elevates TβMCA in bile acid, inhibits FXR signaling pathway [152].

*Note*: ‘+’, protection; ‘‐‘, no protection or no current evidence.

**FIGURE 5:**
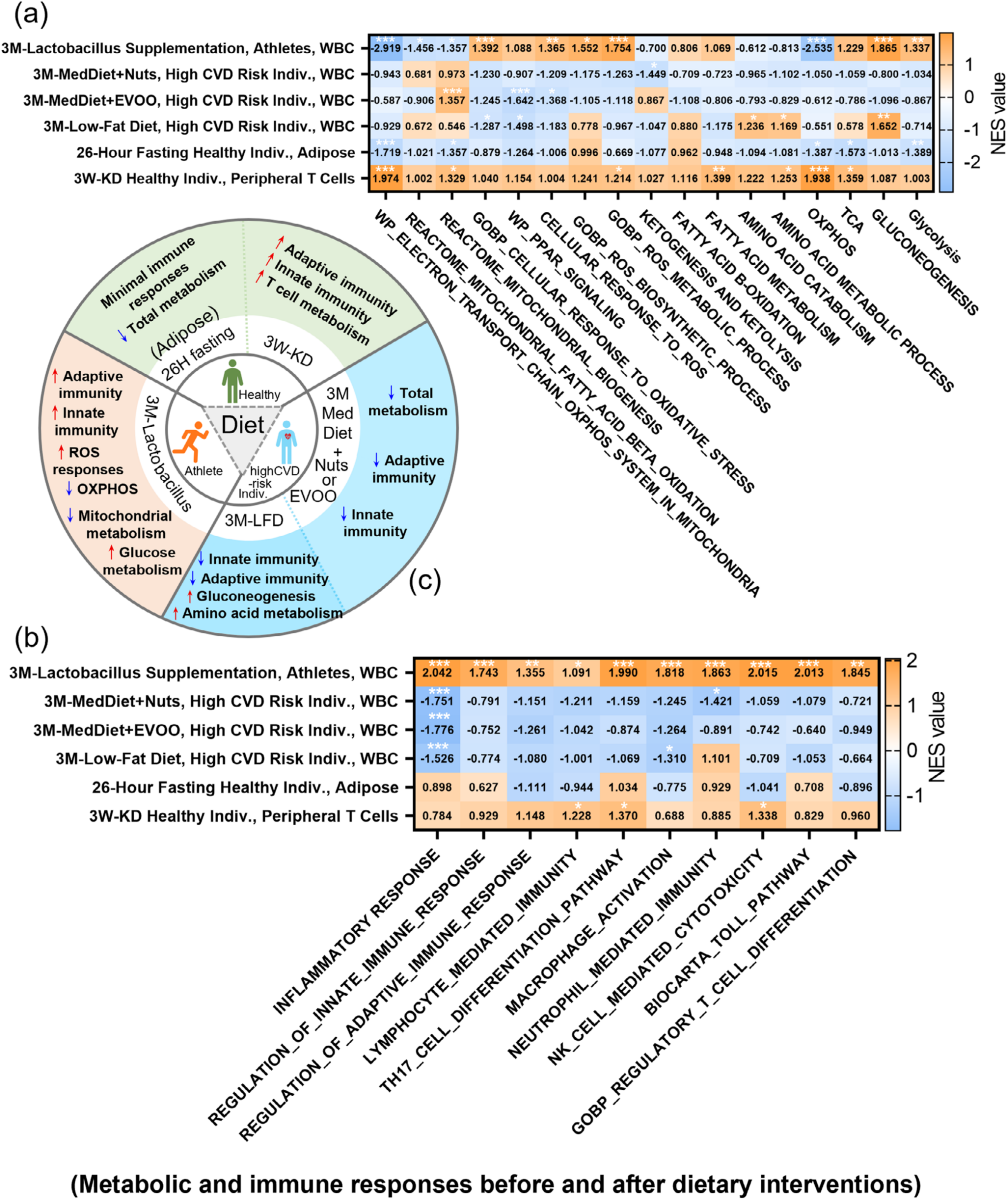
Metabolic and immune phenotype alterations under various dietary interventions. Heatmaps of normalized enrichment scores (NES) from RNA‐seq data for (a) metabolism‐ and (b) immune‐related gene sets in white blood cells (WBCs) of athletes before and after 3 months (3M) of lactobacillus supplementation (n = 44); WBCs of individuals with high cardiovascular disease (CVD) risk before and after 3M of a Mediterranean diet (MedDiet) plus extra virgin olive oil (EVOO) (n = 11), MedDiet plus mixed nuts (n = 11), or a low‐fat diet (n = 9); adipose tissue of healthy individuals after 26‐h fasting diet (n = 11); and peripheral T cells of healthy individuals after 3 weeks (3W) of a ketogenic diet (KD) (n = 28).(c) Schematic diagram summarizing immune and metabolic alterations. The number marked (a, b) is NES value. ^*^, *p* < 0.05; ^**^, *p* < 0.01; ^***^, *p* < 0.001. Data were obtained from GEO (GSE122671, GSE28358, GSE154610, and GSE158407), with gene sets from the Molecular Signatures Database (refer to Excels S1 and S2), and GSEA performed using v4.3.2 (Broad Institute).

The Mediterranean diet, characterized by abundant fruits, vegetables, whole grains, legumes, nuts, and olive oil, is consistently associated with reduced hepatic steatosis, improved insulin sensitivity, and lower fibrosis risk [301, 302]. Mechanistically, it decreases ceramide synthesis, enhances mitochondrial function, reduces hepatic fat accumulation, and enriches beneficial microbial taxa such as *Faecalibacterium prausnitzii* and *Bifidobacterium*, increases SCFA production, and lowers systemic inflammation [300, 301]. When combined with structured exercise, it may further promote fibrosis regression and liver enzyme normalization [303]. In individuals at high cardiovascular risk, Mediterranean diet supplementation with nuts or extra virgin olive oil (EVOO) modulated immune and metabolic pathways in circulating white blood cells (Figure 5), supporting systemic benefits beyond the liver.

Low‐carbohydrate/high‐fat diets (LCHFD) and 5:2 intermittent fasting regimens can reduce steatosis and fibrosis, improve metabolic health, and attenuate inflammation in selected settings, partly through hepatic PPARα and PCK1 pathways that promote fatty acid oxidation and insulin sensitivity [304, 305]. Even a single 26‐h fast suppressed metabolic activity and induced mild immune responses in the adipose tissue of healthy individuals (Figure 5). Low‐fat/high‐carbohydrate diets (LF/HCD) primarily improve steatosis and insulin responsiveness by reducing hepatic lipid accumulation [302], whereas general low‐carbohydrate or low‐fat diets can reduce *de novo* lipogenesis and body weight, providing short‐term improvements in insulin resistance [306]. Additionally, three months of a low‐fat diet can modulate immune and metabolic pathways in individuals with high cardiovascular risk (Figure 5). Together, these effects should be interpreted in the context of baseline metabolic state, adherence, and disease stage.

High‐fiber and plant‐forward diets enrich SCFA‐producing bacteria such as *Parabacteroides distasonis*, *Bacteroides acidifaciens*, *Faecalibacterium prausnitzii*, and *Butyricicoccus*. These microbial shifts improve barrier function, reduce endotoxemia, and exert anti‐inflammatory effects that are particularly relevant in MASH [296, 297, 298, 299]. Fermented food‐rich diets can also enhance microbial diversity and reduce host inflammatory markers [307]. Although direct liver‐specific clinical evidence remains limited, their ability to restore microbial homeostasis, reinforce barrier function, increase anti‐inflammatory metabolites, and dampen inflammatory signaling supports their use as adjunctive strategies [127, 307]. Additional protective effects have been reported for the Dietary Approaches to Stop Hypertension (DASH) diet [308] and omega‐3 fatty acid supplementation [309, 310].

Targeted dietary components also show promise. Low‐protein diets (LPD) may reduce *de novo* lipogenesis and hepatic injury by limiting amino acid substrates for lipogenesis [311]. The EAT‐Lancet reference diet, emphasizing plant‐based foods with reduced red meat, is associated with lower MASLD prevalence and improved metabolic function, mainly through reduced inflammation and hepatic fat [312]. Soluble fibers, such as inulin and oligofructose, can alleviate liver injury by enriching *Bacteroides acidifaciens*, activating FXR‐FGF15 signaling, and improving barrier function [298, 313]. Gluten‐reduced, amylase‐trypsin inhibitor‐free (ATI‐free) diets attenuate steatosis and insulin resistance through TLR4‐mediated suppression of intestinal myeloid cells [314]. Resistant starch lowers intrahepatic triglycerides, circulating LPS, and BCAAs and improves insulin sensitivity independently of weight loss by remodeling the gut microbiota [297]. Stearic acid supplementation reduces steatosis and improves glucose metabolism through *Akkermansia*‐driven bile acid remodeling and FXR signaling suppression [152]. Probiotic supplementation with *Lactobacillus* can modulate immune activation and metabolic pathways, reinforcing the preventive role of microbiota‐directed interventions (Figure 5). Other bioactive nutrients, including dietary fatty acids [152], hyodeoxycholic acid [315, 316], and carotenoid torularhodin [59], have shown protective effects in experimental or translational studies (Table S2).

The ketogenic diet (KD), typically defined by high fat (70%–80%), moderate protein (20%–25%), and very low carbohydrate intake (5%–10%), has received growing attention (Tables 2, 3). KD can improve hepatic steatosis and insulin resistance [317], reduce intestinal inflammation [318], enhance antiviral immunity [319], and inhibit HCC growth by altering ALDOB enzymatic function [239, 303]. However, its effects are complex and context‐dependent. KD can induce p53‐dependent senescence [320], cause gastrointestinal and metabolic side effects [321], and produce inconsistent outcomes in cancer, including metastasis promotion through BACH1‐ATF4 activation in some settings [322]. In liver models, KD has been reported to increase cholesterol, IL‐6, p‐JNK, and insulin resistance, thereby worsening NASH and fibrosis [323]. HCC cells may also exploit ketone bodies for growth [324]. Short‐term KD (three weeks) in healthy individuals induced broad immune and metabolic pathway activation (Figure 5), but these observations do not justify unsupervised use in advanced fibrosis or cancer. KD should therefore be considered cautiously and only under clinical monitoring in patients with chronic liver disease or HCC.

In summary, harmful diets drive chronic liver disease by reducing microbial diversity, altering microbiota composition, and impairing gut‐liver homeostasis. In contrast, diets rich in fiber, prebiotics, probiotics, fermented foods, or plant‐derived unsaturated fats can restore microbial balance, strengthen the intestinal barrier, and reduce inflammation. Mediterranean and high‐fiber dietary patterns currently have the strongest evidence for improving MASLD, MASH, and early fibrosis. More specialized strategies, including intermittent fasting, resistant starch, or selected bioactive nutrients, offer additional opportunities but require long‐term validation. Collectively, dietary interventions act through convergent mechanisms, including reduced hepatic lipid burden, bile acid modulation, improved insulin sensitivity, and inflammation attenuation.

### 6.3 Precision Nutrition Based on Baseline Microbiome and Host Context

Dietary interventions are unlikely to be uniformly effective because baseline microbiome composition, host metabolic state, sex, medication exposure, geography, and disease etiology determine metabolite output and physiological response. Precision nutrition, therefore, requires moving beyond broad diet labels, such as “high fiber” or “Mediterranean diet”, toward measurable functional responses, including SCFA production, bile‐acid conversion, indole synthesis, endotoxin burden, and postprandial glucose and lipid handling [325, 326]. Recent resistant‐starch trials in MASLD illustrate this principle. Although resistant starch reduced intrahepatic triglycerides and improved metabolic parameters in many participants, approximately one‐third of participants showed limited benefit. Multi‐omics analyses linked poor response to baseline *Prevotella*‐dominated communities that inhibited resistant‐starch‐degrading bacteria [297, 327]. Conversely, *Bifidobacterium pseudocatenulatum* strains restored resistant‐starch utilization and improved response, and baseline microbial and clinical features predicted treatment efficacy with clinically meaningful accuracy.

A practical precision‐nutrition framework for chronic liver disease could include [97]: (i) baseline clinical stratification by fibrosis stage, diabetes or obesity status, alcohol intake, viral hepatitis status, sex, menopausal status, medication exposure, and HCC risk; (ii) stool metagenomics to quantify fiber degraders, SCFA producers, bile‐acid‐transforming genes, endotoxin‐producing taxa, and pathobiont expansion; (iii) plasma or fecal metabolomics to measure SCFAs, bile acids, indoles, choline metabolites, and inflammatory lipids; and (iv) adaptive dietary assignment and monitoring. Examples include resistant starch plus Bifidobacterium support for low fermenters, Mediterranean or high‐fiber diets for dysbiosis with low SCFA output, alcohol/fructose restriction for endotoxemia‐driven injury, and cautious avoidance of ketogenic diets in advanced fibrosis or HCC unless clinically supervised. Such stratification would transform diet from a generic recommendation into a microbiome‐informed therapeutic tool.

## 7 Conclusions and Perspectives

The transition from chronic liver injury to HCC is not a single linear pathway but a set of overlapping, etiology‐specific trajectories converging on shared immunometabolic failure. MASLD/MASH provides a powerful model for understanding how lipid overload, insulin resistance, mitochondrial stress, dysbiosis, and chronic inflammation drive fibrosis and tumor immune escape. However, ALD, HBV, HCV, and mixed‐etiology liver disease reach HCC through partially distinct initiating events while still engaging the gut‐liver axis through barrier dysfunction, microbial translocation, altered bile‐acid metabolism, fibrotic remodeling, and immune dysfunction.

These insights support a shift from viewing chronic liver disease as isolated histological entities to viewing it as interconnected immunometabolic states shaped by etiology, genetics, sex, diet, environment, and microbiome composition. Clinically, this systems‐level perspective argues for integrative diagnostics that combine immune phenotyping, circulating metabolites, microbial functional signatures, fibrosis assessment, and spatial or tissue‐based biomarkers when available. Such composite approaches may identify high‐risk patients earlier than any single measurement.

Currently, several questions remain central. First, which patients with early steatosis will progress to MASH, cirrhosis, or HCC, and which will remain stable or regress? Robust predictive markers, including genetic variants, immune signatures, microbial taxa, and/or metabolite profiles, are needed to distinguish benign steatosis from aggressive trajectories. Second, which gut microbes and metabolites causally drive disease rather than simply marking disease severity? Third, which immunometabolic pathways should be targeted at each stage, and how can bile‐acid signaling, barrier repair, metabolic normalization, and immune modulation be combined safely? Fourth, can noninvasive blood or stool panels reliably track progression or therapeutic response without requiring biopsy? Fifth, why do some patients develop HCC without cirrhosis, and do these cases arise from distinct metabolic, immune, microbial, or viral programs?

Future research should therefore integrate etiology, sex, spatial context, and baseline microbiome composition into both mechanistic studies and clinical trials. Longitudinal multi‐omics should combine stool metagenomics, plasma and fecal metabolomics, host transcriptomics, single‐cell and spatial profiling, and epigenomics to define which microbial signals are causal drivers rather than bystanders. Therapeutic development should prioritize mechanism‐guided combinations, such as metabolic normalization plus barrier repair, bile‐acid modulation plus immune checkpoint therapy, or microbiome restoration followed by cellular immunotherapy. Precision nutrition and microbiota‐directed interventions will be most effective when matched to the patient's microbial functional capacity, host metabolic state, and hepatic disease stage.

In conclusion, preventing and treating chronic liver disease will require an integrated framework that connects diet, microbiota, metabolism, immunity, fibrosis, and the hepatic spatial niche. By moving from descriptive associations to causal, cell‐type‐specific, and patient‐stratified mechanisms, the field can develop more precise biomarkers and rational interventions. An integrated gut‐liver‐immune paradigm offers a realistic route to intercept chronic liver disease before malignant transformation and to improve therapy in established HCC.

## Author Contributions

Y. Hu and Y. Z. Wu: Conceptualization, Data curation, Formal analysis, Funding acquisition, Investigation, Methodology, Project administration, Resources, Supervision, Validation, Visualization, Original draft, Review and Editing. C. Y. Lin and L. J. Zhang: Investigation, Methodology, Literature summarization, Project administration, Validation, Visualization. H. W. Li and X. Jiang: Conceptualization, Resources, Validation, or Visualization.

## Funding

This work was supported by the National Natural Science Foundation of China (32270950), the Natural Science Foundation of Guangdong Province of China (2025A1515010628; 2024A1515010551), and the Startup Foundation of the Zhuhai People's Hospital (YNXM20210305).

## Conflicts of Interest

The authors declare no conflicts of interest.

## Supporting information




**Supporting File 1**: advs76514‐sup‐0001‐SuppMat.docx.

**Supporting File 2**: advs76514‐sup‐0002‐DataFile.zip.

## Data Availability

Data are provided within the manuscript or supplementary information files.
