# Supplementary material for: Gut Microbiota, Immunity, and Metabolism in the Progression From Chronic Liver Disease to Hepatocellular Carcinoma
Source: Adv Sci (Weinh). 2026 Jul 10;13(44):e23582. doi: 10.1002/advs.202523582 (PMC13353184; doi:10.1002/advs.202523582)
Supplement: Supplementary file 1 [file ADVS-13-e23582-s002.docx]

**Supplemental Tables**

**Table S1.** Summaries of drugs, supplementations, and active ingredients of herbs that have been reported to ameliorate liver disease.

| **Drugs,**  **Supplementations,**  **Herb bioactives** | **MASLD** | **MASH** | **Fibrosis** | **Cirrhosis** | **HCC** | **Conclusions** | **Key mechanisms** |
| --- | --- | --- | --- | --- | --- | --- | --- |
| **Semaglutide** | - | + | - | - | - | Ameliorates steatosis, MASH, inflammation, delays fibrosis progression, improves insulin resistance and metabolic syndrome, and promotes weight loss. | Activates the GLP-1 receptor signaling. ^[1,[2]^ |
| **Tirzepatide** | - | + | + | - | - | Improves MASH, reduces liver fibrosis, lowers liver enzymes, improves metabolic parameters, promotes weight loss. | Activates GIP, GLP-1 receptors. ^[3]^ |
| **Statin** | + | + | + | + | +  - | Reduces hepatic steatosis,slows liver fibrosis progression, reduces risks of hepatocellular carcinoma and hepatic decompensation. | Inhibits HMG-CoA reductase, reduce expressions of TGFβ1, CTGF, PDGFβ. ^[4,[5]^ |
| **Vitamin E (300mg/day)** | - | + | + | - | - | Reduces steatosis, lobular inflammation, and fibrosis, decreases liver enzyme levels and proinflammatory cytokines. | Reduces oxidative stress and hepatocyte damage. ^[6]^ |
| **Vitamin B12 +folate** | - | + | + | - | - | Alleviates MASH by reducing inflammation and fibrosis, restoring autophagy, decreasing homocysteine levels, and improving lipid metabolism. | Converts homocysteine to methionine, prevents homo-cysteinylation and ubiquitination of STX17. ^[7]^ |
| **Bempedoic acid** | + | + | - | - | - | Alleviates diet-induced hepatic steatosis, promotes fatty acid oxidation, improves glucose tolerance, and reduces liver lipid accumulation through an ACLY-independent mechanism. | Activates PPARα signaling to promote fatty acid oxidation gene expression. ^[6]^ |
| **Caffeine** | + | + | + | - | - | Ameliorates MASH in mice by rescuing hepatic Dusp9 expression and inhibiting the ASK1-p38/JNK signaling pathway. | Upregulates hepatic Dusp9, which inactivates the ASK1-p38/JNK signaling pathway. ^[8]^ |
| **Glutamate** | - | - | + | + | + | Enhances liver regeneration, improves liver function, accelerates repair after injury, reduces fibrosis, and increases hepatocyte proliferation. | Hepatocytic URII–GS–HIF1α/WNT3–YAP1 regulatory axis governing macrophage metabolic reprogramming and liver regeneration. ^[9]^ |
| **Tripeptide (Gly-Gly-L-Leu, DT-109)** | + | + | + | - | - | Glycine-based treatment reduces hepatic lipid accumulation, enhances fatty acid oxidation, promotes glutathione synthesis, alleviates oxidative stress and inflammation, and reduce gut Clostridium sensu stricto. | Stimulates hepatic fatty acid β-oxidation and de novo glutathione synthesis, reduces oxidative stress, inhibits NF-κB and TGFβ/SMAD signaling. ^[10]^ |
| **Linghe granules** | + | - | - | - | - | Linghe (苓荷) granules (multi-herbal formulation) reduce hepatic lipid accumulation, improve liver function, decrease oxidative stress, modulate lipid metabolism. | Regulates SREBP-1, SCD1, FABP5, Nrf2, STAT3, and GPX4. ^[11]^ |
| **Torularhodin** | + | - | - | - | - | Reduces hepatic lipid accumulation, improves dyslipidemia, modulates gut microbiota, enhances gut barrier function, inhibits ceramide accumulation. | Enriched Akkermansia muciniphila promotes adenosylcobalamin synthesis, which inhibits the HIF-2α pathway, reduces ceramide levels. ^[12]^ |
| **Cordycepin** | + | + | + | - | - | Reduces hepatic lipid accumulation, suppresses inflammation, ameliorates liver injury, attenuates fibrosis, lowers liver enzymes, and improves lipid metabolism. | Activates AMPK signaling pathway, which inhibits lipid synthesis and NF-κB pathways. ^[13]^ |
| **Salidroside** | - | - | + | - | - | Ameliorates overtraining-induced liver fibrosis, overtraining-related hepatic fibrosis by reducing muscle lactate body formation. | SORBS3-lactylation & FBXO2 signal activate BAX/BAK to induce hepatocyte MCL1 degradation and apoptosis. ^[14]^ |
| **Hyperoside** | + | - | - | - | - | Alleviates hepatic steatosis, reduces liver lipid deposition, improves lipid metabolism, exerts anti-inflammatory effects, modulates gut microbiota, increases conjugated bile acids, and reduces bile acid-induced hepatotoxicity. | Activates FXR and inhibits ACLY to regulate bile acid and fatty acid metabolism. ^[15]^ |
| **Gastrodin** | + | + | + | - | - | Reduces hepatic lipid accumulation, suppresses inflammation, attenuates fibrosis, improves insulin resistance and metabolic syndrome. | Activated AMPK signaling downregulates lipogenic genes (e.g., FASN, SCD1, ACC), enhances fatty acid oxidation, inhibits NF-κB. ^[16]^ |
| **Alnustone** | + | + | + | - | - | Reduces hepatic lipid accumulation, improves insulin resistance, anti-inflammatory, reverses liver fibrosis, enhances mitochondrial function, lowers liver injury, improves lipid metabolism, reduces hepatocyte apoptosis. | Increases Ca²⁺ signals, mitochondrial fatty acid β-oxidation. ^[17]^ |
| **Hyperoside** | + | - | - | - | - | Reduces hepatic steatosis, decreases cholesterol and triglyceride accumulation, promotes bile acid synthesis and excretion, inhibits lipogenesis, enhances fatty acid oxidation, reduces inflammation, and improves lipid metabolism. | FXR/LXRα- CYP7A1/CYP27A1 axis promotes cholesterol conversion to bile acids, inhibits SREBP-1/ACC-mediated lipogenesis. ^[18]^ |
| **Carabrone** | + | + | - | - | - | Attenuates hepatic steatosis, reduces inflammation and fibrosis, improves insulin resistance and glucose metabolism, decreases lipid accumulation. | Inhibits STAT3 activation, reducing lipid accumulation, and inflammation. ^[19]^ |
| **Taxifolin（TAX）** | - | - | + | - | - | Attenuates liver fibrosis, reduces hepatocyte apoptosis and DNA damage, suppresses inflammation, inhibits HSC activation, and may delay fibrosis progression via targeting pNDRG1T328. | binds to NDRG1 at Cys289, inhibits its phosphorylation at Thr328, suppresses SGK1–NDRG1 interaction. ^[20]^ |
| **Nuciferine** | + | - | - | - | - | Alleviates hepatic steatosis, improves insulin resistance, reduces oxidative stress and inflammatory response, activates the autophagy-lysosomal pathway. | Disrupts Rag-HBXIP interaction, inhibits mTORC1 activity, activates the TFEB-mediated autophagy-lysosomal pathway. ^[21]^ |
| **Buddleoside** | + | + | + | - | - | Alleviates hepatic steatosis, improves insulin sensitivity, reduces inflammation and fibrosis, activates autophagy. | Targets PRKAB1 subunit of AMPK (at Val81, Arg83, Ser108), which phosphorylates RPTOR to inhibit mTORC1, leading to TFEB nuclear translocation and activation of the autophagy-lysosomal pathway, thereby ameliorating MASH. ^[22]^ |
| **Cyclovirobuxine D** | - | - | - | - | + | Induces mitophagy, promotes cell cycle arrest, reduces oncogenic signaling, inhibits tumor growth and metastasis, suppresses HCC progression. | Targets LIF at Val145, activates p38MAPK/p62, induces mitophagy. ^[23]^ |
| **Berberine** | - | - | - | - | + | Inhibits tumor progression, modulates tumor immune microenvironment, enhances T cell function, regulates cytokine secretion, and promotes immune cell communication. | Increases central memory CD8^+^ T cells and thereby enhances antitumor immunity against HCC. ^[24]^ |
| **Ponicidin** | - | - | - | - | + | Inhibits HCC cell proliferation, promotes tumor cell apoptosis, induces mitochondrial dysfunction, increases ROS production, downregulates anti-apoptotic proteins, suppresses migration and invasion. | Targets Keap1-PGAM5 complex, promoting ubiquitination and degradation of PGAM5, which activates the caspase-dependent mitochondrial apoptosis pathway. ^[25]^ |

**Table S2.** Representative clinical trials of diets alleviate development and progression of liver disease.

| **Diseases** | **NCT** | **Status** | **Intervention** | **Recruit number** | **Start date** | **Location** | **Clinical outcome** |
| --- | --- | --- | --- | --- | --- | --- | --- |
| **MASLD** | NCT01650025 | Completed | VSL#3, a mixture of eight probiotic strains for 4 months | 48 | Aug.  2012 | Italy ^[26]^ | Beneficial |
|  | ISRCTN05474560 | Completed | VSL#3, a mixture of eight probiotic strains for 10 weeks | 35 | Aug.  2012 | UK ^[27]^ |  |
| **MASH** | NCT04822181 | Not recruiting | Semaglutide vs. Placebo | 1205 | Apr,  2021 | USA etc 408 locations ^[1]^ | Beneficial |
|  | NCT02970942 | Completed |  | 320 | Nov,  2016 | USA etc 158 locations ^[2]^ |  |
|  | NCT04166773 |  | Tirzepatide vs. Placebo | 190 | Nov,  2019 | USA etc 112 locations ^[3]^ |  |
|  | NCT05073588 |  | Indo Mediterranean diet vs. Calorie restricted diet | 39 | Aug,  2021 | India ^[28]^ |  |
|  | NCT04283942 |  | Intermittent calorie restriction (ICR) vs.  Continuous calorie restriction (CCR) | 60 | July,  2020 | China ^[29]^ |  |
|  | NCT01798719 |  | Low Glycemic Index Mediterranean Diet vs.  General Advice | 50 | Feb,  2011 | Italy ^[30]^ |  |
|  | NCT03118310 |  | 5:2 fasting diet vs. Low-Carb High-Fat Diet vs. Placebo diet | 74 | Apr,  2017 | Sweden ^[31]^ |  |
|  | NCT04322110 |  | PronoKal Method vs. Low calorie diet | 40 | Sept,  2016 | - ^[32]^ |  |
|  | NCT02568605 |  | Prebiotic fibre vs. Placebo | 45 | May,  2015 | Canada ^[33]^ |  |
|  | NCT04066400 |  | Nutritional Intervention | 40 | Oct,  2018 | Germany ^[34]^ |  |
|  | NCT00870012 |  | Four strains of *Lactobabillus* & *Bifidobacterium* supplementation | 20 | Feb.,  2009 | China |  |
|  | NCT04442620 | Unknown | Physical activity and Mediterranean Diet (PA-MD) vs. High meal frequency of Mediterranean Diet (HMF-MD) vs. Control diet (CD) | 150 | Oct,  2017 | Spain ^[35]^ | **Non** |
|  | NCT03737071 | Not recruiting | Low Carbohydrate Diet vs. Simple Calorie Restriction Diet | 10 | Nov,  2018 | Finland ^[36]^ | **Non** |
| **Fibrosis** | NCT06308757 | Recruiting | Very-low-calorie ketogenic diet (VLCKD) with meal replacements vs. Mediterranean low-calorie diet (LCD) | 42 | Sept,  2021 | Italy | **Non** |
|  | NCT06845345 |  | Mediterranean Intermittent Fasting Diet vs. Medication | 70 | Apr,  2025 | Netherlands | **Non** |
|  | NCT04175392 | Terminated | Align Probiotic Supplement Capsule vs. Placebos | 14 | Jan,  2021 | USA | **Non** |
|  | NCT02021253 | Completed | Probiotic vs. Placebo | 64 | Apr,  2013 | France ^[37]^ | **Non** |
|  | NCT03863730 | Not recruiting | ReFerm^®^+FSMP+probiotics vs. Fresubin (dietary supplement) | 56 | Mar,  2019 | Denmark | **Non** |
| **Cirrhosis** | NCT01135628 | Completed | Hyperproteic and fiber-rich diet vs. Nitazoxanide | 60 | Aug,  2010 | Mexico | **Non** |
|  | NCT01603199 |  | High protein high fiber diet | 36 | Sept,  2011 | Mexico | **Non** |
|  | NCT01655121 |  | High protein high fiber diet | 40 | Jan,  2012 | Mexico | **Non** |
|  | NCT06425380 |  | Resistant Potato Starch | 11 | July,  2024 | USA | **Non** |
|  | NCT02418039 | Terminated | High protein diet vs. Normal protein | 42 | Mar,  2017 | Mexico | **Non** |
|  | NCT07028580 | Recruiting | Diet based on animal proteins vs. Diet based on plant proteins vs. General instructions for patients with liver disease. | 100 | June,  2022 | Athens, Attica, Greece | **Non** |
|  | NCT06464952 |  | Resistant potato starch vs. Powdered cellulose | 30 | July,  2024 | USA | **Non** |
| **HCC** | NCT07143955 | Completed | Structured Dietary Intervention (high-fiber, lean protein) vs. Routine Dietary Care | 100 | May,  2022 | China | **Non** |
|  | NCT02438436 |  | Simo decoction vs. Gum chewing | 180 | Mar,  2015 | China | **Non** |
|  | NCT03853928 | Unknown status | Probiotics (*Lactobacillus*, *Lactobacillus plantarum*, *Streptococcus faecalis*, *Bifidobacterium*) | 280 | May,  2019 | Argentina | **Non** |
|  | NCT07064668 | Not recruiting | TUDCA (Tauroursodeoxycholic Acid) Supplementation vs. Immune checkpoint inhibitor (ICI) | 300 | July,  2025 | China | **Non** |

Note: Non, not known.

References

1.	Sanyal, A.J., et al., *Phase 3 Trial of Semaglutide in Metabolic Dysfunction–Associated Steatohepatitis.* New England Journal of Medicine, 2025. **392**(21): p. 2089-2099.

2.	Newsome, P.N., et al., *A Placebo-Controlled Trial of Subcutaneous Semaglutide in Nonalcoholic Steatohepatitis.* New England Journal of Medicine, 2021. **384**(12): p. 1113-1124.

3.	Loomba, R., et al., *Tirzepatide for Metabolic Dysfunction–Associated Steatohepatitis with Liver Fibrosis.* New England Journal of Medicine, 2024. **391**(4): p. 299-310.

4.	Choi, J., et al., *Statin Use and Risk of Hepatocellular Carcinoma and Liver Fibrosis in Chronic Liver Disease.* JAMA Internal Medicine, 2025. **185**(5).

5.	Pustjens, J., et al., *TOP-457-YI Statin, but not aspirin use, is inversely associated with steatotic liver disease and liver fibrosis: results from two large population based studies.* Journal of Hepatology, 2025. **82**: p. S622-S623.

6.	Liu, J.Y., et al., *Bempedoic acid suppresses diet-induced hepatic steatosis independently of ATP-citrate lyase.* Cell Metabolism, 2025. **37**(1): p. 239-254.e7.

7.	Tripathi, M., et al., *Vitamin B12 and folate decrease inflammation and fibrosis in NASH by preventing syntaxin 17 homocysteinylation.* Journal of Hepatology, 2022. **77**(5): p. 1246-1255.

8.	Xin, X., et al., *Caffeine ameliorates metabolic-associated steatohepatitis by rescuing hepatic Dusp9.* Redox Biology, 2025. **80**.

9.	Rigual, M.d.M., et al., *Macrophages harness hepatocyte glutamate to boost liver regeneration.* Nature, 2025. **641**(8064): p. 1005-1016.

10.	Rom, O., et al., *Glycine-based treatment ameliorates NAFLD by modulating fatty acid oxidation, glutathione synthesis, and the gut microbiome.* Science Translational Medicine, 2020. **12**(572).

11.	Hu, Y., et al., *Linghe granules reduces hepatic lipid accumulation in Non-alcoholic fatty liver disease through regulating lipid metabolism and redox balance.* Phytomedicine, 2025. **141**.

12.	Liu, C., et al., *The carotenoid torularhodin alleviates NAFLD by promoting Akkermanisa muniniphila-mediated adenosylcobalamin metabolism.* Nature Communications, 2025. **16**(1).

13.	Lan, T., et al., *Cordycepin Ameliorates Nonalcoholic Steatohepatitis by Activation of the AMP‐Activated Protein Kinase Signaling Pathway.* Hepatology, 2021. **74**(2): p. 686-703.

14.	Liu, Y., et al., *Muscle-derived small extracellular vesicles induce liver fibrosis during overtraining.* Cell Metabolism, 2025. **37**(4): p. 824-841.e8.

15.	Wang, S., et al., *Hyperoside modulates bile acid and fatty acid metabolism, presenting a potentially promising treatment for non-alcoholic fatty liver disease.* Journal of Advanced Research, 2025.

16.	Wan, J., et al., *Gastrodin Improves Nonalcoholic Fatty Liver Disease Through Activation of the Adenosine Monophosphate–Activated Protein Kinase Signaling Pathway.* Hepatology, 2021. **74**(6): p. 3074-3090.

17.	Hu, S., et al., *Alnustone Ameliorates Metabolic Dysfunction‐Associated Steatotic Liver Disease by Facilitating Mitochondrial Fatty Acid β‐Oxidation via Targeting Calmodulin.* Advanced Science, 2025.

18.	Wang, S., et al., *Hyperoside attenuates non-alcoholic fatty liver disease in rats via cholesterol metabolism and bile acid metabolism.* Journal of Advanced Research, 2021. **34**: p. 109-122.

19.	Pan, A., et al., *Carabrone Attenuates Metabolic Dysfunction–Associated Steatohepatitis by Targeting STAT3 in Mice.* MedComm, 2025. **6**(3).

20.	Ding, C., et al., *Taxifolin attenuates liver fibrosis by regulating the phosphorylation of NDRG1 at Thr328 via hepatocyte-stellate cell cross talk.* Acta Pharmaceutica Sinica B, 2025. **15**(4): p. 2059-2076.

21.	Du, X., et al., *Nuciferine protects against high-fat diet-induced hepatic steatosis and insulin resistance via activating TFEB-mediated autophagy–lysosomal pathway.* Acta Pharmaceutica Sinica B, 2022. **12**(6): p. 2869-2886.

22.	Chen, M., et al., *Buddleoside alleviates nonalcoholic steatohepatitis by targeting the AMPK-TFEB signaling pathway.* Autophagy, 2025. **21**(6): p. 1316-1334.

23.	Shao, Y., et al., *Targeting LIF With Cyclovirobuxine D to Suppress Tumor Progression via LIF/p38MAPK/p62‐Modulated Mitophagy in Hepatocellular Carcinoma.* MedComm, 2025. **6**(6).

24.	Hu, J., et al., *Berberine Protects against Hepatocellular Carcinoma Progression by Regulating Intrahepatic T Cell Heterogeneity.* Advanced Science, 2024. **11**(39).

25.	Zhao, B., et al., *Ponicidin Promotes Hepatocellular Carcinoma Mitochondrial Apoptosis by Stabilizing Keap1‐PGAM5 Complex.* Advanced Science, 2024. **11**(38).

26.	Alisi, A., et al., *Randomised clinical trial: The beneficial effects of VSL#3 in obese children with non-alcoholic steatohepatitis.* Aliment Pharmacol Ther, 2014. **39**(11): p. 1276-85.

27.	Chong, P.L., et al., *A randomised placebo controlled trial of VSL#3((R)) probiotic on biomarkers of cardiovascular risk and liver injury in non-alcoholic fatty liver disease.* BMC Gastroenterol, 2021. **21**(1): p. 144.

28.	Deshmukh, A., et al., *Effect of Indo‐Mediterranean diet versus calorie‐restricted diet in children with non‐alcoholic fatty liver disease: A pilot randomized control trial.* Pediatric Obesity, 2024. **19**(11).

29.	Sun, X., et al., *Intermittent compared with continuous calorie restriction for treatment of metabolic dysfunction-associated steatotic liver disease: a randomized clinical trial.* The American Journal of Clinical Nutrition, 2025. **121**(1): p. 158-166.

30.	Misciagna, G., et al., *Effect of a low glycemic index Mediterranean diet on non-alcoholic fatty liver disease. A randomized controlled clinici trial.* The Journal of nutrition, health and aging, 2017. **21**(4): p. 404-412.

31.	Holmer, M., et al., *Treatment of NAFLD with intermittent calorie restriction or low-carb high-fat diet – a randomised controlled trial.* JHEP Reports, 2021. **3**(3).

32.	Cunha, G.M., et al., *Efficacy of a 2-Month Very Low-Calorie Ketogenic Diet (VLCKD) Compared to a Standard Low-Calorie Diet in Reducing Visceral and Liver Fat Accumulation in Patients With Obesity.* Frontiers in Endocrinology, 2020. **11**.

33.	Mayengbam, S., et al., *Effects of combined prebiotic fiber supplementation and weight loss counseling in adults with metabolic dysfunction-associated steatotic liver disease: a randomized controlled trial.* European Journal of Nutrition, 2025. **64**(4).

34.	Armandi, A., et al., *Short‐term reduction of dietary gluten improves metabolic‐dysfunction associated steatotic liver disease: A randomised, controlled proof‐of‐concept study.* Alimentary Pharmacology & Therapeutics, 2024. **59**(10): p. 1212-1222.

35.	Quetglas-Llabrés, M.M., et al., *Mediterranean Diet Improves Plasma Biomarkers Related to Oxidative Stress and Inflammatory Process in Patients with Non-Alcoholic Fatty Liver Disease.* Antioxidants, 2023. **12**(4).

36.	Luukkonen, P.K., et al., *Effect of a ketogenic diet on hepatic steatosis and hepatic mitochondrial metabolism in nonalcoholic fatty liver disease.* Proceedings of the National Academy of Sciences, 2020. **117**(13): p. 7347-7354.

37.	Roussel, E., et al., *Influence of Probiotics Administration Before Liver Resection in Patients with Liver Disease: A Randomized Controlled Trial.* World Journal of Surgery, 2021. **46**(3): p. 656-665.
